# Human Y Chromosome Exerts Pleiotropic Effects on Susceptibility to Atherosclerosis

**DOI:** 10.1161/ATVBAHA.119.312405

**Published:** 2019-09-05

**Authors:** James M. Eales, Akhlaq A. Maan, Xiaoguang Xu, Tom Michoel, Pille Hallast, Chiara Batini, Daniel Zadik, Priscilla R. Prestes, Elsa Molina, Matthew Denniff, Juliane Schroeder, Johan L.M. Bjorkegren, John Thompson, Pasquale Maffia, Tomasz J. Guzik, Bernard Keavney, Mark A. Jobling, Nilesh J. Samani, Fadi J. Charchar, Maciej Tomaszewski

**Affiliations:** 1 From the Division of Cardiovascular Sciences, Faculty of Biology, Medicine and Health, University of Manchester, United Kingdom (J.M.E., A.A.M., X.X., B.K., M.T.); 2 The Roslin Institute, The University of Edinburgh, United Kingdom (T.M.); 3 Computational Biology Unit and Department of Informatics, University of Bergen, Norway (T.M.); 4 Institute of Biomedicine and Translational Medicine, University of Tartu, Estonia (P.H.); 5 Department of Health Sciences (C.B., J.T.), University of Leicester, United Kingdom; 6 Department of Genetics and Genome Biology (D.Z., M.A.J.), University of Leicester, United Kingdom; 7 Department of Cardiovascular Sciences (M.D., N.J.S., F.J.C.), University of Leicester, United Kingdom; 8 School of Health and Life Sciences, Federation University Australia, Ballarat, Victoria (P.R.P., E.M., F.J.C.); 9 Centre for Immunobiology, Institute of Infection, Immunity and Inflammation (J.S., P.M.), College of Medical, Veterinary and Life Sciences, University of Glasgow, United Kingdom; 10 Institute of Cardiovascular and Medical Sciences (J.S., P.M., T.J.G.), College of Medical, Veterinary and Life Sciences, University of Glasgow, United Kingdom; 11 Department of Genetics and Genomic Sciences, Icahn Institute for Genomics and Multiscale Biology, Icahn School of Medicine at Mount Sinai, New York, NY (J.L.M.B.); 12 Department of Pharmacy, University of Naples Federico II, Italy (P.M.); 13 Jagiellonian University College of Medicine, Kraków, Poland (T.J.G.); 14 Division of Medicine, Manchester University NHS Foundation Trust, Manchester Academic Health Science Centre, United Kingdom (B.K., M.T.); 15 NIHR Leicester Biomedical Research Centre, United Kingdom (N.J.S.); 16 Department of Physiology, University of Melbourne, Parkville, Victoria, Australia (F.J.C.).

**Keywords:** coronary artery disease, epigenomics, gene expression, humans, risk factors

## Abstract

The male-specific region of the Y chromosome (MSY) remains one of the most unexplored regions of the genome. We sought to examine how the genetic variants of the MSY influence male susceptibility to coronary artery disease (CAD) and atherosclerosis.

**Approach and Results:**

Analysis of 129 133 men from UK Biobank revealed that only one of 7 common MSY haplogroups (haplogroup I1) was associated with CAD—carriers of haplogroup I1 had ≈11% increase in risk of CAD when compared with all other haplogroups combined (odds ratio, 1.11; 95% CI, 1.04–1.18; *P*=6.8×10^−4^). Targeted MSY sequencing uncovered 235 variants exclusive to this haplogroup. The haplogroup I1–specific variants showed 2.45- and 1.56-fold respective enrichment for promoter and enhancer chromatin states, in cells/tissues relevant to atherosclerosis, when compared with other MSY variants. Gene set enrichment analysis in CAD-relevant tissues showed that haplogroup I1 was associated with changes in pathways responsible for early and late stages of atherosclerosis development including defence against pathogens, immunity, oxidative phosphorylation, mitochondrial respiration, lipids, coagulation, and extracellular matrix remodeling. *UTY* was the only Y chromosome gene whose blood expression was associated with haplogroup I1. Experimental reduction of *UTY* expression in macrophages led to changes in expression of 59 pathways (28 of which overlapped with those associated with haplogroup I1) and a significant reduction in the immune costimulatory signal.

**Conclusions:**

Haplogroup I1 is enriched for regulatory chromatin variants in numerous cells of relevance to CAD and increases cardiovascular risk through proatherosclerotic reprogramming of the transcriptome, partly through *UTY*.

## Abstract 2

Supplemental Digital Content is available in the text.

**Highlights**

- Y chromosome haplogroup I1 is associated with an increased risk of coronary artery disease.
- Genetic variants specific to haplogroup I1 show an enrichment in overlap with transcription start site and enhancer chromatin states in epigenomes from cells and tissues relevant to coronary artery disease.
- We have identified gene expression pathways involved in both early and late stages of atherosclerosis, which are perturbed in men who inherited haplogroup I1 from their fathers.
- The expression of one of Y chromosome genes (*UTY*) is reduced in blood samples from men with haplogroup I1 when compared with those with other haplogroups.
- Experimental reduction in expression of *UTY* leads to perturbation of pathways of gene expression related to atherosclerosis and a significant reduction in abundance of the costimulatory proteins CD80 and CD83 in induced macrophages.

Transmitted as an indivisible portion of DNA from fathers to sons, the male-specific region of the Y chromosome (MSY) constitutes 95% of the entire length of the human Y chromosome.^1^ Since the divergence of both sex chromosomes from a pair of autosomes 300 million years ago, the gene content of the MSY has been significantly reduced, and the Y is now one of the smallest chromosomes with the lowest gene content.^2^ However, recent studies revealed that mammalian evolution fixed at least a handful of ubiquitously expressed genes on MSY because of their apparent importance to male viability and survival.^3^

**See accompanying editorial on page 2201**

The MSY does not routinely recombine or exchange genetic material with any other elements of the genome.^4,5^ Because of its haploid nature, the MSY has been routinely excluded from genome-wide association studies.^4^ As a result, there is a gap in knowledge on how the genetic variation within the human Y chromosome contributes to male health and susceptibility to disease. Our previous analysis of MSY showed that one of its common European lineages, haplogroup I, was associated with increased risk of coronary artery disease (CAD) when compared with other haplogroups.^5^ This association was independent of conventional cardiovascular risk factors.^5^ Our further transcriptome-wide analysis of human macrophages revealed that a majority of autosomal gene sets showing differential expression between haplogroup I and other MSY lineages map to immune and inflammatory pathways.^5^ Collectively, these data suggest that the MSY genetically regulates susceptibility to CAD, possibly through modulation of the immune response. This adds to an expanding body of evidence from experimental animal models and human studies suggesting that genetic variation within the Y chromosome plays an important role in male health.^6^

Here, through a phylogenetic analysis of 129 133 Y chromosomes, we uncover a new association between one of the MSY lineages (haplogroup I1) and CAD. Through DNA sequencing of the MSY combined with analysis of chromatin state across 21 epigenomes, we further demonstrate that this paternal lineage of the Y chromosome is enriched for genetic variants with a functional effect on gene expression in cells and tissues of relevance to atherosclerosis. Our transcriptome-wide analyses in tissues of relevance to CAD show that haplogroup I1 carriers exhibit changes in gene expression in numerous pathways known for their role in atherosclerosis—immunity, response to viral infection, energy and lipid metabolism, coagulation, and hemostasis, as well as extracellular remodeling. Finally, our studies implicate a single *MSY* gene whose experimentally reduced expression in human macrophages leads to changes in expression of genes and pathways partly replicating those under the genetic control of haplogroup I1.

## Materials and Methods

### Association Between Y Chromosome Haplogroups and CAD

#### UK Biobank—Y Chromosome Genotyping

UK Biobank utilized 2 genotyping arrays: ≈50 000 individuals were genotyped on UK BiLEVE array, and the remaining individuals on the UK Biobank Axiom array.^7^ These 2 arrays harbor 813 and 1041 single-nucleotide variants of the Y chromosome, respectively. At the time of our analyses, genetic data were available for 223 513 male individuals. A series of stringent quality control (QC) procedures were undertaken at both the genotype and sample level (Figures I and II in the online-only Data Supplement). Four metrics were used as a QC check for the Y chromosome variants on each array (Figure I in the online-only Data Supplement). These included (1) applying UK Biobank internal variant QC, (2) overlap with International Society of Genetic Genealogy (ISOGG; www.isogg.org) variants, (3) exclusion of variants with low call rate, and (4) exclusion of monomorphic variants. A total of 162 Y chromosome variants survived the QC analysis and were used for the purpose of haplogroup analysis. A separate QC pipeline was used to exclude samples of insufficient quality before the association analysis (Figure II in the online-only Data Supplement). These included assessment of genotype missingness rate, heterozygosity, familial relatedness, genetic sex, and genetic ancestry. The number of samples excluded at each stage of the QC process together with the number of surviving samples are shown in Figure II in the online-only Data Supplement. Of 169 648 individuals surviving the QC filters, 168 686 had matching fully informative Y chromosome haplogroup information.

#### UK Biobank—Phylogenetic Analysis

**Figure 1.**
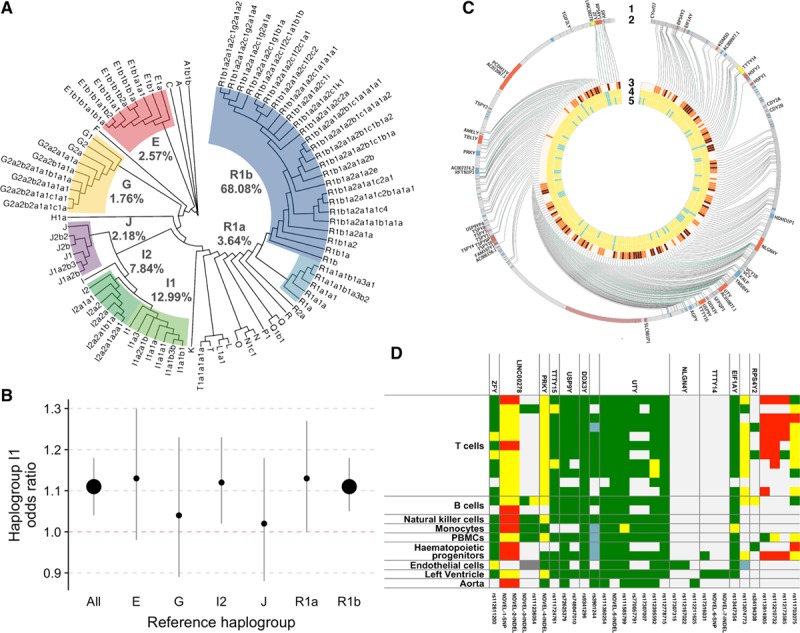
The Y chromosome and coronary artery disease. **A**, Phylogenetic analysis of the Y chromosome in UK Biobank. Colored (matched to Hallast et al) groups of tree branches examined for association with coronary artery disease (CAD) and their respective frequencies. **B**, Odds ratios for CAD in men with haplogroup I1 compared with men with other haplogroups of the Y chromosome. Error bars show the lower and upper bounds of the 95% CI. The size of the point reflects the level of statistical significance after correction for multiple testing (associations that survived the correction for multiple testing are shown as large points). **C**, Two hundred thirty-five genetic variants specific to haplogroup I1 uncovered through DNA sequencing of male-specific region of the Y chromosome (MSY). Variant locations are visualized as radial lines (2) originating in the outermost gray circle in the context of known protein-coding genes (red boxes), noncoding genes (yellow), and pseudogenes (blue boxes) of the Y chromosome; gene symbols are external to the outermost circle (1). Links to the inner circles are colored in green for variants mapping to genes and gray for intergenic variants. The number of datasets in which each variant appears (3) is scaled from light yellow (1) to dark red (3). Single-nucleotide substitutions are shown in yellow and insertion-deletions in turquoise (4). Novel variants are also highlighted in turquoise (5). **D**, Thirty-one genetic variants exclusive to haplogroup I1 shown in context of the summarized Roadmap Epigenomics chromatin states from 21 epigenomes of relevance to atherosclerosis/CAD. Individual epigenomes are grouped under the same type of cell/tissue. Red denotes a transcription start site state; yellow, an enhancer state; green, a transcribed state; and pale gray, a silent state. *MSY* gene symbols are annotated above the heat map variant identifier (wherever present).

Using genotype call data for Y chromosome variants that had passed QC, PED and MAP files were generated as input to the yHaplo^8^ software—custom-designed software for Y chromosome haplogroup assignment. In brief, yHaplo^8^ uses a reference phylogenetic tree structure with haplogroup-defining single-nucleotide polymorphisms (SNPs) that is regularly updated to reflect refinements by ISOGG. For each individual, the algorithm traverses through the branches of the phylogenetic tree according to SNP calls, starting at the root and stopping when the most terminal branch possible is reached.^8^ A total of 85 MSY haplogroups were identified by yHaplo (see Figure 1A; Table I in the online-only Data Supplement). Of those, 7 groupings (E, G, I1, I2, J, R1a, and R1b) within the top level of the MSY phylogenetic tree were present in >1% of men.

#### UK Biobank—CAD Phenotyping

CAD was defined using hard end point criteria derived from self-assessment, hospital episode statistics, and death registry data in the UK Biobank cohort. Patients with CAD (cases) were defined as (1) men with either self-reported or hospital episode statistics-documented history of myocardial infarction or coronary revascularization procedures, including percutaneous coronary intervention or coronary artery bypass graft and (2) men whose death had been attributed to CAD in death registry data. CAD-free controls were defined as men with no history of angina, unstable angina, myocardial infarction, coronary revascularization, death due to CAD or taking common CAD medications (aspirin, glyceryl trinitrate, isosorbide mononitrate/dinitrate, and nicorandil) across self-reported, hospital episode statistics, and death registry records. A total of 11 234 men with CAD and 117 899 CAD-free male controls were identified using these criteria (as illustrated in detail in a diagnostic decision tree developed for the purpose of this project [Figure III in the online-only Data Supplement]) and had full information required for adjustment for cofounders (Table II in the online-only Data Supplement).

#### UK Biobank—Genetic Association Analyses

For the purpose of our genetic association analysis, we selected all common (>1%) haplogroups identified through our phylogenetic analysis (E, G, I1, I2, J, R1a, and R1b). The association analysis was conducted by multiple logistic regression with CAD as the dependent variable and binarized haplogroup status of the Y chromosome (ie, E versus others), age, body mass index, genotyping array, hypertension, physical activity level, household income, smoking, completion of further education, employment status, alcohol intake frequency, paternal and maternal history of heart disease, and 5 genetic principal components extracted from autosomal genotypes were independent variables (Tables II through IX in the online-only Data Supplement). Similar to other genetic studies on individuals of white British ancestry,^9^ we adopted the first 5 principal components in our regression models to account for effects of hidden population structure. These components are the first 5 of 40 principal components generated in UK Biobank using 147 604 autosomal single-nucleotide variants in 407 219 biologically unrelated individuals of different ethnicities.^7^ Given 7 multivariate logistic regression models constructed in our genetic association analysis, the Bonferroni correction for multiple testing was applied at *P*=0.0071 (0.05/7). For the purpose of validation, we conducted an additional analysis of association between haplogroup I1 and CAD using a different set of diagnostic criteria of CAD, as applied to UK Biobank by Klarin et al.^10^ This analysis of association was conducted using the same statistical model and the same set of covariates as specified earlier.

### Next-Generation DNA Sequencing of Haplogroup I1

We combined data from 3 separate DNA-sequencing experiments to obtain the most extensive list of genetic variants associated with haplogroup I1: (1) de novo targeted sequencing of MSY regions with the strongest potential of biological functionality, (2) previously conducted targeted high-coverage sequencing of 3.7 Mb of MSY DNA,^11,12^ (3) low-coverage whole genome sequencing in the 1000 Genomes Project.^13,14^

#### De Novo Targeted MSY DNA Sequencing

##### Individuals

Forty-eight white British individuals were selected from those recruited previously.^15^ Individuals were selected on the basis of genotyping data for key UK haplogroup-defining markers including M170 for haplogroup I and M269 for R1b1b2 haplogroups. Of the selected individuals, 15 men were confirmed as carriers of haplogroup I1 by genotype comparison of 5 different haplogroup I1 defining single-nucleotide variants from the ISOGG reference set.

##### Sequencing Targets

###### Genes.

Using Ensembl (release 75) as a reference, we designed amplicons to cover all exons, introns, and 3′- and 5′-untranslated regions (together with 100-bp adjacent downstream and upstream sequences) of all known protein-coding and noncoding genes on the MSY (coding status defined by Ensembl attribute gene_biotype). Amplicons were screened by the Agilent HaloPlex SureDesign service to remove amplicons covering highly repetitive DNA (using balanced amplicon selection; Table X in the online-only Data Supplement).

###### Transcription Factor–Binding Site Targets.

Through an analysis of Encyclopedia of DNA Elements (ENCODE) chromatin immunoprecipitation sequencing (ChIP-seq) data, we identified 522 putative transcription factor–binding sites. Putative transcription factor–binding sites were extracted from ENCODE ChIP-seq peak calling outputs (peakSeq) for all available tissues and cell types and across all transcription factors available. BigBed files generated by peakSeq were first converted to bed and then filtered for peaks on chrY; these were then sorted by chromosomal position and merged using bedtools resulting in 522 putative sites that were then extended to a minimum of 500 bp. Amplicons covering the sites were created and screened for repetitiveness by Agilent SureDesign (using maximum-specificity amplicon selection; Table X in the online-only Data Supplement).

###### Novel Transcribed Regions.

Analysis of RNA-sequencing data from monocyte and macrophage cell isolates (samples prepared as in the study by Schunkert et al^16^) identified 8 regions of the Y chromosome that show signs of active transcription but are not covered by gene annotations in Ensembl. Reads were aligned to the Ensembl GRCh37 genome (using the transcriptome reference gtf, Ensembl, version 75) using TopHat. Intergenic regions containing >10 gapped reads and with mapping quality >20 were included as targets for sequencing.

###### Haplogrouping SNPs.

We also targeted 2972 Y chromosome haplogrouping SNPs identified from 3 sources: the ISOGG SNP index (https://isogg.org/tree/ISOGG_YDNA_SNP_Index.html), the variant list from Wei et al,^17^ and the Illumina HumanExome genotyping array (HumanExome-12-v1-2-B). Haplogrouping SNPs were sorted by chromosomal position and merged (to remove duplicate SNPs).

The full set of 3648 MSY target regions gained an amplicon coverage of 78%, covering 3665 kb of MSY sequence (Table X in the online-only Data Supplement). The summary characteristics for the sequenced targets are shown in Table X in the online-only Data Supplement.

##### DNA Sequencing

The targeted regions were selected and enriched for using an Agilent Haloplex kit (with adapter ligation for Illumina sequencers). The enrichment process was performed at the Oxford Genomics Centre at the Wellcome Trust Centre for Human Genetics. Input to the Haloplex process was 225 ng of genomic DNA (extracted from peripheral blood). Sequencing libraries with Illumina adapters were constructed as part of the Haloplex protocol. All libraries were simultaneously sequenced across 2 lanes of a high-output run on an Illumina HiSeq2000 using 100-bp paired-end reads.

##### Read QC and Mapping

Raw reads first underwent QC checks including manual inspection of per sequence, per tile, and per base read quality distributions, as well as sequencing adapter content and per base sequence content by FastQC (https://www.bioinformatics.babraham.ac.uk/projects/fastqc/) run with the --nogroup argument. Raw reads were trimmed using trimmomatic^18^ in paired-end mode with the input arguments SLIDINGWINDOW:5:20, MINLEN:35, and ILLUMINACLIP:adapters.fa:3:15:3:1:true. Reads were mapped to the GRCh37 human genome reference using Bowtie2 with default arguments.

##### Variant Calling

A total of 5.12 Gbp of MSY-mapped read data from 15 men with haplogroup I1 underwent variant calling using the GATK HaplotypeCaller (GATK v4^19^). HaplotypeCaller was set to a ploidy of one and was limited to 3 alternative alleles per variant site. We applied a set of variant filtering criteria using the GATK tool (v4), VariantFiltration, all filter specific criteria are outlined in Table XI in the online-only Data Supplement. To test the stability of our variant calling process, we screened for false-positive and false-negative variants using a DNA sample from the 1000 Genomes Project, the same individual had undergone Complete Genomics whole genome sequencing, a high-quality custom genome sequencing process that produces variant calls with a high level of reliability. A comparison of our variants calls with the Complete Genomics set did not identify a single instance of false-positive or false-negative variant calls. A total of 1463 unique variants including 1112 SNPs and 351 insertion-deletions were called and retained for further analysis.

#### High-Coverage Sequencing of MSY

##### Individuals

A total of 448 men representing a worldwide spectrum of MSY phylogeny underwent high-coverage (≈51×) sequencing of 3.7 Mb of MSY DNA.^11,12^ From this dataset, we selected 4 men of European ancestry who were identified as carriers of haplogroup I1 based on haplogroups from Hallast et al.^12^

##### Library Preparation, Sequencing, and QC

Full experimental details are available elsewhere.^11,12^ In brief, 3 to 5 µg of genomic DNA extracted from peripheral blood was used for library preparation and SureSelect (Agilent Technologies) target enrichment. The samples were sequenced with paired-end 100-bp reads on an Illumina HiSeq2000. All reads were mapped to the GRCh37 human genome reference, subjected to stringent quality filters and comparison to complete genomics reference genotypes for a set of 1000 Genomes reference DNA samples.

##### Variant Calling

From the whole dataset, we obtained 1.59 Gbp of aligned and filtered reads from 4 men with haplogroup I1. Variant calling was conducted using identical methods to those above. A total of 1202 unique variants including 1037 SNPs and 165 insertion-deletions were called and retained for further analysis.

#### MSY Variants From Phase 3 of the 1000 Genomes Project

##### Individuals

A total of 31 individuals with haplogroup I1 were selected from the 1000 Genomes Project (phase 3 release)^13,14^ for the purpose of this analysis. We selected these individuals based on haplogroup inferences made as part of the 1000 Genomes Project phase 3^14^.

##### Variant Calls

Variants for each haplogroup I1 individual were selected from the phase 3 variant call format files for the Y chromosome (ftp://ftp.1000genomes.ebi.ac.uk/vol1/ftp/release/20130502/ALL.chrY.phase3_integrated_v2a.20130502.genotypes.vcf.gz). We excluded copy number variants from the analysis (ie, an alternative allele of “<CN0>” or “<CN1>”). We checked the specificity of our haplogroup I1–specific variants by comparing all variants shared by all our haplogroup I1 samples against those found in all other 1000 Genomes Project samples; any overlapping variants were excluded.

#### Combination of MSY Variants From 3 Experiments

We combined all haplogroup I1 MSY variant calls from the 3 experiments into a single master variant call set by merging them on GRCh37 start and end position, as well as reference and alternative alleles using R. We also recorded the presence (or absence) of every variant in each of the 3 datasets.

### Functional Analysis of Haplogroup I1–Specific Variants in Cells and Tissues of Relevance to Atherosclerosis and CAD

#### Summarization of Roadmap Epigenomics 15-State Chromatin Segmentation

The Roadmap Epigenomics 15-state chromatin segmentation data^20^ labels human genome with one of 15 possible chromatin states^20^ across 127 epigenomes derived from human tissues and cell lines. Of those, we selected 21 epigenomes (Table XII in the online-only Data Supplement) based on their known relevance to atherosclerosis or CAD. We grouped the 15 chromatin states into 4 states using a combination of histone mark specificity (Table XIII in the online-only Data Supplement) and the 15-state clustering reported by Roadmap Epigenomics in their extended data.^20^ In brief, states 1 to 3 were combined into transcription start site (“Tss”; based on H3K4me4), states 4 and 5 into “transcribed” (based on H3K36me3), and states 6 and 7 into “enhancer” (H3K4me1); the remainder were combined into a “silent/other” state.

**Figure 2.**
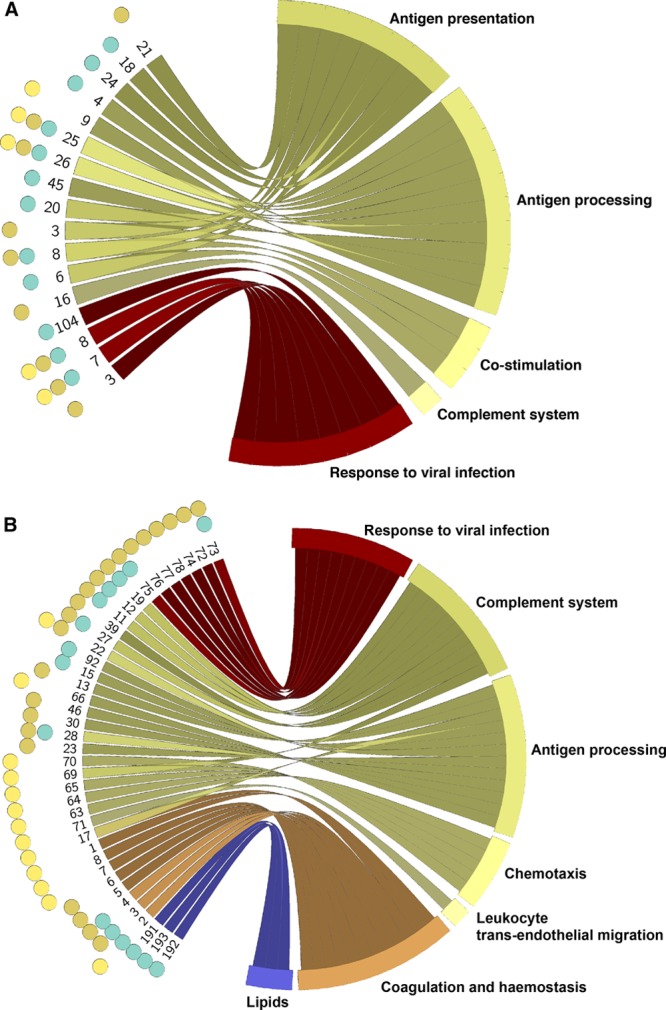
Biological pathways of gene expression modified in men from haplogroup I1 when compared with other haplogroups. Genotype-Tissue Expression (GTEx) project (**A**) and STAGE (Stockholm Atherosclerosis Gene Expression; **B**) results are shown separately. Tissues where a given pathway is associated with haplogroup I1 are shown as colored points; yellow, heart; brown, adipose tissue; and turquoise, arterial tissue. Pathways are numbered as per Tables XXII (GTEx) and XXIV in the online-only Data Supplement (STAGE). Colored links associate pathways with specific biological themes; color is determined by the functional theme, and each theme is labeled.

#### Mapping Genetic Variants to Chromatin Regions With Functional Potential

We computed the overlapping chromatin state for each identified haplogroup I1 variant in each of the 21 epigenomes using GRCh37 start and end positions for each variant, allowing for a variant to overlap with >1 state (within a single epigenome).

#### Summarized Active Chromatin State Enrichment Analysis

The chromatin states for the identified 235 haplogroup I1 variants were compared with all common (those with at least 1% minor allele frequency) nonhaplogroup I1 MSY variants from European 1000 Genomes Project phase 3 individuals. Further sensitivity analyses were restricted to 216 haplogroup I1 variants present in the 1000 Genomes Project (phase 3 release) and compared with the same MSY reference (all European MSY variants with at least 1% minor allele frequency). The *P* for enrichment in active chromatin states in haplogroup I1 variants in both the atherosclerosis/CAD-relevant set and for other epigenomes was calculated by 1 million random permutation of the haplogroup I1 variants; for each permutation, the fold enrichment is calculated and compared with the fold change for nonpermuted variants. The final *P* was calculated by counting the number of permutations where the nonpermuted enrichment value is exceeded divided by the number of permutations performed.

To compare the enrichment in active chromatin states for haplogroup I1 variants between atherosclerosis/CAD-relevant and other epigenomes, we generated 1 million random permutations of epigenomes not included in the atherosclerosis/CAD-relevant set and calculated the fold enrichment between I1 and common MSY SNPs for each state in each permutation. A *P* for each chromatin state was calculated by counting the number of permuted fold-enrichment values that exceeded the fold enrichment in CAD-relevant epigenomes divided by the number of permutations performed.

For the Genotype-Tissue Expression (GTEx) project eSNP comparison, we first identified 2 Roadmap epigenomes that had a pair in the GTEx V6 eQTL analysis output (aorta and left ventricle, https://www.gtexportal.org/home/datasets). We selected the GTEx results for each tissue to only include the most statistically significant eSNPs (identified by is_chosen_snp=1). This identified 4417 SNPs for left ventricle and 6220 for aorta. As a comparator to the GTEx eSNPs, we used common SNPs from the dbSNP common_all data set (build 150); this includes SNPs with minor allele frequency >1% in at least one of 26 major human populations. We removed all known eSNPs (identified by GTEx v6) from dbSNP before any comparisons were made, leaving 37 436 593 SNPs for the analysis.

### Pathway Analysis of Gene Expression Data From GTEx

#### MSY Genotypes and Haplogrouping of GTEx Men

Called genotypes (in variant call format) for each male GTEx individual genotyped by Illumina DNA sequencing were downloaded from dbGaP (database of genotypes and phenotypes). We excluded individuals with <80% call rate of Y chromosome markers; this yielded 178 male individuals suitable for phylogenetic analysis. Genotypes for M170 (a marker for haplogroup I) and M253 (a marker for haplogroup I1) were extracted and compared with ISOGG data to identify haplogroup informative genotypes to classify each individual as I1 (having genotypes M170=C and M253=T) or other (having genotypes M253=C).

#### Principal Components

Three principal components were derived from autosomal genotype data generated by the GTEx project using genotypes from the Illumina Omni 5M array and compared between carriers of haplogroup I1 and men with other lineages of the Y chromosome using a Kolmogorov-Smirnov test.

#### Differential Gene Expression Analysis

The GTEx read count table (release V6p) was downloaded from the GTEx portal. Read count data for every tissue of each haplogrouped sample were extracted. As recommended by GTEx, samples with unreliable transcriptome information were removed (subject phenotype field SMTORMVE=FLAGGED), leaving 173 male individuals with informative read count data. We tested each of 8 different tissues of relevance to CAD (Table XIV in the online-only Data Supplement)—for differential expression between men with haplogroup I1 versus men who are carriers of other haplogroups. Differential expression analysis was conducted by DESeq2^21,22^ with GTEx read counts as an input. The analysis was adjusted for age, body mass index, sample ischemic time, sample sequencing center, and a variable number of surrogate variables. The number of surrogate variables calculated per tissue was 10% of the number of samples tested. Surrogate variables were calculated by SVASeq^23^ on the middle 80% of expressed genes in each tissue. The top and bottom 10% were excluded to reduce the impact of highly and weakly expressed genes,^23^ which are more likely to generate artifactual read count values. Outlier read count observations (per gene) were identified and replaced by DESeq2^21,22^ using Cook distances and the trimmed mean of read counts per gene. In all differential expression analyses, MSY haplogroup was binarized into “I1” and “other” values, with other set as the reference level. Thus, all differential expression and pathway analysis outputs show deviation of haplogroup I1 expression from other haplogroups, with a positive coefficient (effect size) indicative of higher expression in men with haplogroup I1 than among others. Using the full output from DESeq2, we then extracted the T statistic for every tested gene, and these were provided as input to our pathway analysis.

#### Pathway Analysis

Our pathway analysis followed the gene set enrichment analysis (GSEA) methodology.^24^ This approach to transcriptome analysis can detect subtle differences in expression of predefined biologically coordinated gene sets from the Molecular Signatures DataBase embedded in the KEGG, BIOCARTA, and REACTOME pathway repositories. Our analysis was conducted on tissues of relevance to atherosclerosis/CAD between men with haplogroup I1 and those from other haplogroups (as reported above). The pathway analysis testing process was performed by the R package fgsea on the canonical pathways gene set from Molecular Signatures DataBase (v5.1 c2.cp.v5.1.symbols.gmt) with a million permutations on 10 processors. Input was the distinct set of HGNC (HUGO gene nomenclature committee) gene symbols with their associated T statistics from the DESeq2 differential expression testing process. Significant pathways were identified by having a Benjamini-Hochberg (false discovery rate)–adjusted *P*<0.05. A positive normalized enrichment score corresponds to increased expression of leading edge genes within a given pathway in carriers of haplogroup I1. Immunity subthemes, themes, and immune-relevant genes were determined by 2 independent experts in immunology.

### Pathway Analysis of Gene Expression Data From the STAGE Study

#### STAGE Cohort Characteristics

The STAGE study (Stockholm Atherosclerosis Gene Expression) collected a set of 612 tissue samples from Swedish individuals.^25^ Gene expression and genotype data have been derived for each sample using the custom Affymetrix HuRSTA-2a520709 and the Affymetrix GenomeWideSNP_6 arrays, respectively. For the purpose of this analysis, we selected atherosclerotic artery wall (AAW), subcutaneous fat (SF), visceral fat (VF), whole blood (WB) gene expression profiles from a total of 92 men with available genotyping information (Table XV in the online-only Data Supplement). We included STAGE men in this project (1) to establish an independent replication for the findings from GTEx (2) to capture the potential differences between the compared haplogroups in pathways that may be quiescent in nondiseased tissues.

#### MSY Genotypes and Haplogrouping of STAGE Men

From the STAGE genotypes we extracted biallelic genotype calls for the 9 different haplogroup I1–specific markers recommended by the ISOGG database. Men whose alleles were consistent with the haplogroup I1–indicative allele across all markers were classified as carriers of haplogroup I1. We observed that the frequency of haplogroup I1 in STAGE is much higher (≈35%; Table XV in the online-only Data Supplement) than in GTEx (Table XIV in the online-only Data Supplement); this is in line with previously published estimates of prevalence of haplogroup I1 in Swedish population.^25^

#### Principal Components

We calculated the first 3 genetic principal components using the methods of the GTEx consortium^26^ on genotype data from the Affymetrix GenomeWideSNP_6 array. Principal component values were compared between carriers of haplogroup I1 and men with other lineages of the Y chromosome using a Kolmogorov-Smirnov test.

#### Differential Gene Expression Analysis

STAGE gene expression data were background corrected and normalized using the Affymetrix GeneChip command console. Normalized probe intensities were examined for differential expression between I1 and other MSY haplogroups using linear regression adjusting for age and body mass index, with other set as the reference level. In consistency with the differential expression analysis conducted in GTEx, we extracted the gene symbols and T statistics as input to the pathway analysis.

#### Pathway Analysis

The pathway analysis for each STAGE tissue was performed in the same way as in the GTEx analysis. Immunity subthemes, themes, and immunity-relevant genes were determined by 2 independent experts in immunology.

#### Sensitivity Analysis

A further differential gene expression analysis was conducted with additional adjustment for clinically relevant parameters including total cholesterol, HDL (high-density lipoprotein) cholesterol, hypertension, and treatment with lipid-lowering medication available in STAGE. In consistency with the primary analysis, gene symbols and T statistics from the differential gene expression analysis were used as input to the pathway analysis. All significant pathways from the primary analysis were retested by GSEA and the statistics compared between primary and the secondary sensitivity analysis (Table XVI in the online-only Data Supplement). All significant results from the primary analysis produced the same direction of pathway association (identical sign of the normalized enrichment score), 388 of 389 results replicated at a nominal *P* of 0.05 and 386 results (of 389) replicated at false discovery rate of 5%.

### Experimental Reduction of *UTY* Gene Expression in Human Macrophages

#### Generation and Isolation of Macrophages

Peripheral blood mononuclear cells were isolated from a healthy male donor’s whole blood sample obtained from the Australian Red Cross, West Melbourne. Monocytes were converted to macrophages using a recombinant human macrophage colony-stimulating factor–based technique reported in detail previously.^5^

#### UTY Knockdown by Antisense Oligonucleotides

We used advanced antisense oligonucleotides Locked Nucleic Acid longRNA GapmeRs—a class of high-affinity RNA analogues that exhibit high thermal stability when hybridized to a cDNA or RNA strand—to reduce expression of *UTY* (ubiquitously transcribed tetratricopeptide repeat containing, Y linked). Antisense Locked Nucleic Acid longRNA GapmeRs were designed and synthesized by Exiqon. The transfection of the following GapmeRs, UTY_GapmeR (product sequence 5′-3′: AATGGAGTGTCTGTGC), and negative control A (product sequence 5′-3′: AACACGTCTATACGC) were performed using Lipofectamine 2000 (Thermo Fisher Scientific) following Exiqon manufacturer’s recommendations. Cells were seeded into 6-well plates at a density of ≈2×105/mL and cultured in DMEM supplemented with 10% HI-FBS, penicillin (100 U/mL), and streptomycin (100 μg/mL) until cells reached ≈70% confluence. Subsequently, cells were transfected with 10 nmol/L of antisense oligonucleotides in Lipofectamine 2000 reagent.

#### RNA Extraction and RNA Analyses

Forty-eight hours post-transfection, total RNA was extracted using the TRIzol reagent followed by a RNA cleanup step using miRNeasy kit (Qiagen). The knockdown was confirmed by real-time quantitative polymerase chain reaction. The latter was performed using SYBR Green (Bioline) according to the manufacturer’s instructions. In brief, 20 ng (2 μL) of cDNA was added to 2.5 μL of SYBR Green qPCR reaction mix (Bioline) together with 200 nmol/L of each of the forward and reverse primer (Integrated DNA Technologies) and 0.3 μL of nuclease-free water in a final volume of 5 μL. We used the following *UTY* primers (forward primer, 5′-TCTACAGAATGGTTCTGATAACTGGAA-3′; reverse primer, 5′-GGTGTCAAACACAACGAATAAACTTG-3′) and the following primers for the control gene (GAPDH; forward primer, 5′-CTCTGCTCCTCCTGTTCGAC-3; reverse primer, 5′-GCGCCCAATACGACCAAATC-3′). Amplification and detection were conducted under the following conditions: 10 minutes at 95°C, 45 cycles of 15 s at 95°C and 1 minute at 60°C on Applied Biosystems ViiA 7 Real-Time PCR system (Life Technologies).

For the purpose of RNA sequencing, the libraries were constructed using 3 g of input macrophage RNA using the Illumina TruSeq RNA protocol with poly-A selection. Libraries were sequenced using 100-bp reads (on an Illumina HiSeq 2000) producing an average of 26.3 million reads and 2.63 Gb per sample. All generated raw reads were stored in FASTQ format. The base call and read quality were evaluated using FastQC (https://www.bioinformatics.babraham.ac.uk/projects/fastqc/). The input library complexity was assessed using RNA-SeQC.^27^ cDNA, sequencing libraries, and raw RNA-sequencing data were generated at the Australian Genome Research Facility.

#### Differential Gene Expression Analysis and Pathway Analyses

Gene expression was quantified in all samples using Kallisto^28^ and a transcriptome index built from Ensembl GRCh38 release 83 with bias correction enabled, and 100 bootstrap quantifications were performed. Read counts were examined for differential expression between knockdown and control (with control as reference level) samples in the Sleuth R package29 using all 100 bootstrap replicates and default parameters. Differentially expressed genes were identified by a Wald test^29^ at the significance threshold of false discovery rate <0.05. Similar to studies in GTEx and STAGE, we extracted T statistics for all genes tested as an input to further GSEA-based pathway analysis. All GSEA analyses were performed in agreement with the principles highlighted earlier.

All in vitro experiments were performed in triplicate across 2 experimental conditions *UTY* knockdown and control.

### Experimental Reduction of *UTY* Gene Expression in THP-1 Cells

#### Cell Culture and Transfection

THP-1 cells were seeded into 24-well plates at 5×105 cells in complete RPMI (10% fetal calf serum, 1% penicillin/streptomycin, and 1% L-glutamine) and differentiated to macrophages with the addition of phorbol 12-myristate 13-acetate (10 ng/mL) for 48 hours. Adherent cells were washed 3× with PBS to remove phorbol 12-myristate 13-acetate and subsequently allowed to rest in complete RPMI for 24 hours before transfection. Cells were transfected with either 5 pmol *UTY* GapmeR (product sequence 5′-3′: AATGGAGTGTCTGTGC; Qiagen), negative control A (product sequence 5′-3′: AACACGTCTATACGC; Qiagen), or Block-iT Fluorescent Oligo (Invitrogen) as transfection control, using the Lipofectamine RNAiMAX Reagent (Invitrogen) following manufacturer’s instructions. To induce macrophage activation, lipopolysaccharide from *Escherichia coli* 0111:B4 (100 ng/mL; Sigma) was added to the cultures. Cells were incubated at 37°C, 5% CO_2_ for 48 hours. Five experimental replicates of each control and knockdown transfection were performed.

#### Flow Cytometry

Cells were harvested and washed in PBS. After blocking Fcγ receptors (5% mouse serum and 0.001% sodium azide in 2.4G2 cell culture supernatant) for 10 minutes at 4°C, cells were stained for 30 minutes with the following anti-human antibodies: CD14 (Pe-Cy7) expressed on macrophages, CD80 (FITC), CD83 (APC), and HLA-DR (e450) or their respective isotype controls (all Thermofisher UK). Unbound antibody was washed off, and cells have been stained for viability using Fixable Viability Dye eFluor 780 (eBioscience). Events have been acquired using the LSR2 and Fortessa flow cytometers (BD Bioscience) and analyzed using FlowJo (Tree Star) software. The differences between 2 contrasting experimental conditions were examined by a Mann-Whitney *U* test.

The studies adhered to the Declaration of Helsinki and were approved/ratified by the NHS National Research Ethics Service North West (United Kingdom), the Leicestershire Health Authority Ethics Committee (United Kingdom), and the Ethics Committee of Karolinska University Hospital (Sweden). Informed written consent was obtained from all recruited individuals.

## Results

### The Main Northern European Lineage of Haplogroup I (Haplogroup I1) Is Associated With Increased Risk of CAD

A total of 162 single-nucleotide variants of the Y chromosome survived the QC filters and were informative in the phylogenetic analysis (Figure I in the online-only Data Supplement). Of 225 516 men recruited in UK Biobank, 168 686 individuals of white European ancestry had MSY genotype information sufficient for the resolution of their Y chromosome haplogroup. Based on this information, we identified 85 distinct haplogroups (Figure 1A), 7 groupings of which were present in at least 1% of the UK population (Figure 1A). A total of 129 133 men classified into one of 7 common haplogroups had full phenotypic information required for the genetic association analysis (Figure II in the online-only Data Supplement; Table II in the online-only Data Supplement). Using the diagnostic decision algorithm, we classified them into individuals with CAD (cases, 11 234) and CAD-free controls (controls, 117 899; Figure III in the online-only Data Supplement). Their clinical characteristics are shown in Table XVII in the online-only Data Supplement. Only one of 7 examined MSY haplogroups was related to CAD after adjusting for its risk factors, genetic principal components, and genotyping array (Figure 1B; Tables III through IX in the online-only Data Supplement). Indeed, haplogroup I1 (most common in Northern Europe) was coupled with ≈11% excess of CAD risk when compared with other MSY lineages (odds ratio, 1.11; 95% CI, 1.04–1.18; *P*=6.8×10^−4^; Figure 1B; Table III in the online-only Data Supplement). We validated the association between haplogroup I1 and CAD (Table XVIII in the online-only Data Supplement) using a different set of diagnostic criteria of CAD applied previously to UK Biobank by Klarin et al.^10^ Further studies conducted on all pairwise combinations of the 7 haplogroups confirmed that statistically significant associations with CAD were apparent only in the analyses of haplogroup I1 (Figure 1B; Table XIX in the online-only Data Supplement). They also revealed a consistency in the direction of effect of haplogroup I1 on the risk of CAD irrespective of which other haplogroup was used as a comparator (Table XIX in the online-only Data Supplement). No statistically significant differences in the risk of CAD were detected between any pair of haplogroups other than I1 (Table XIX in the online-only Data Supplement).

Collectively, in the largest population of men to date with a resolved MSY phylogeny, we identified the association between CAD and haplogroup I1 and showed that other common Y chromosome lineages do not predispose their carriers to increased CAD risk.

### Two Hundred Thirty-Five Genetic Variants Track Exclusively With Haplogroup I1 Along the Paternal Line—Next-Generation DNA Sequencing of MSY

To identify variants that are inherited exclusively from haplogroup I1, we compared DNA profiles of 50 haplogroup I1 chromosomes against all other Y chromosome lineages using data from 3 separate DNA-sequencing experiments covering 10.4 Mb (44%) of the euchromatic portion of the MSY. This analysis revealed 235 variants unique to carriers of haplogroup I1 (Figure 1C; Table XX in the online-only Data Supplement). Of those, 228 (97%) were SNPs and 7 (3%) were insertion-deletions. The identified haplogroup I1–specific variants are dispersed throughout the euchromatic MSY with one polymorphic site per 44.2 kb of the sequenced region on average. Fifty (21%) variants are present within a total of 23 known MSY genes with 26 (52%) of these variants internal to the 15 single-copy “X-degenerate” genes of the Y chromosome. None of the identified haplogroup I1–specific variants were within translated exons of MSY protein-coding genes (Figure 1C; Table XX in the online-only Data Supplement).

Taken together, these experiments showed that although a high number of variants are inherited together as haplogroup I1, none of them appear to act through changes in amino acid sequence of MSY proteins.

### Genetic Variants Exclusive to Haplogroup I1 Are Enriched for Active Chromatin States in Cells and Tissues of Relevance to Atherosclerosis

To further functionally characterize haplogroup I1–specific variants, we first mapped them onto chromatin regulatory elements derived from a core set of 5 histone modifications in Roadmap Epigenomics.^20^ Specifically, we examined an overlap between each of 235 identified variants and 4 combinatorial chromatin states (Figure 1D; Table XIII in the online-only Data Supplement), including transcription start site (“Tss”), enhancer (“Enh”), transcribed (“Tx”), and “silent/other” across 21 epigenomes derived from cells/tissues of relevance to atherosclerosis/CAD. Of 4935 annotations (235 variants by 21 epigenomes), 393 (8%) haplogroup I1–specific annotations overlapped with at least one active (associated with expressed genes) state^20^ in the set of examined cell/tissues (Figure 1D). Forty, 79, and 274 of these annotations were classified as “Tss” (associated with promoter regions), “Enh” (associated with enhancer regions), and “Tx” associated with transcribed regions, respectively (Figure 1D).

Using the same chromatin segmentation states across 21 epigenomes of atherosclerosis-relevant cells/tissues, we then determined the enrichment for active states between variants specific to haplogroup I1 versus any other MSY genetic variants (with at least 1% frequency in European populations). Given the demonstrated association between CAD and haplogroup I1, we have hypothesized that it may be enriched for variants that overlap active chromatin states in cell types/tissues of relevance to atherosclerosis. This analysis demonstrated that haplogroup I1–specific variants show 2.45- and 1.56-fold enrichment for active chromatin states associated with promoters and enhancers in cells/tissues of relevance to atherosclerosis when compared with nonhaplogroup I1 MSY variants (*P*=1×10^−6^ and *P*=1.15×10^−4^), respectively. To exclude a potential detection bias (driven by targeted sequencing of potentially functional regions of MSY in haplogroup I1 carriers), we conducted further sensitivity analyses using 1000 Genomes Project data as an exclusive source of information on MSY variants for all haplogroups. These sensitivity analyses confirmed the enrichment for Tss and enhancer categories for haplogroup I1 when compared with other MSY variants (*P*=1.0×10^−4^ and *P*=1.39×10^−2^), respectively. Finally, we examined whether haplogroup I1 variants are enriched for active chromatin states only in CAD-relevant epigenomes or whether this is also seen in other epigenomes not related to atherosclerosis/CAD. We used a million permutations of our chromatin state data from epigenomes that were not included in the atherosclerosis/CAD-relevant set and found no evidence of enrichment for promoters (0.85-fold; *P*=0.9880) but a statistically significant enrichment for enhancers (1.70-fold; *P*=5×10^−6^) when comparing I1-specific variants with common MSY variants. We have also determined, by a further 1 million permutations, that the enrichment for promoter states is specific to atherosclerosis epigenomes when compared with nonatherosclerosis epigenomes (*P*=2×10^−6^) but that this is not the case for the enhancer state (*P*=0.6530).

To provide global genomic context to the magnitude of haplogroup I1–specific enrichment for active chromatin states, we compared all known expression quantitative loci (eSNPs) identified by GTEx in 2 relevant tissues against non-eSNP autosomal variants (with minor allele frequency >1%) using the same active chromatin states in 2 overlapping Roadmap Epigenomics tissues. eSNPs show a similar (to variants of haplogroup I1) magnitude of enrichment for active chromatin states (“Tss”: 3.02-fold enrichment, *P*=3.1×10^−5^; “Enh”: 1.25-fold enrichment, *P*=2.3×10^−5^).

Taken together, these results show that the Y chromosome sequence of haplogroup I1 is enriched for variants overlapping with the 2 most active chromatin states that regulate gene expression in human cells and tissues and that the magnitude of this enrichment is similar to that of eSNPs.

### Haplogroup I1 Is Associated With Changes in Numerous Pathways of Relevance to Atherosclerosis in Men From the General Population

Using MSY genotypes, we divided GTEx men into carriers of haplogroup I1 and other lineages, respectively (Table XIV in the online-only Data Supplement). There were no statistically significant differences in 3 genetic principal components between the groups (Table XXI in the online-only Data Supplement). We then examined differences in gene expression sets between these 2 groups using RNA sequencing–derived transcriptome profiles for 8 tissues of relevance to atherosclerosis/CAD (online-only Data Supplement). A total of 104 gene sets showed significant association with haplogroup I1 in at least one of 8 GTEx tissues (Figure IV in the online-only Data Supplement; Table XXII in the online-only Data Supplement). Based on prior knowledge of function of differentially expressed leading edge genes, we clustered the haplogroup I1–associated gene sets into 13 overarching biological themes bringing together individual pathways implicated in GSEA under common biological processes. A total of 28 individual pathways were related to immunity; this was identified as the dominant theme underpinning the signature of haplogroup I1 in tissues of relevance to atherosclerosis/CAD. The pattern of immune gene expression implicated through GSEA was consistent with the inhibition of MHC class I and II–restricted antigen presentation, antigen processing, reduced costimulation, and upregulation of the complement system in men with haplogroup I1 (Figure 2A; Table XXII in the online-only Data Supplement). Our GSEA also revealed the association between haplogroup I1 and several pathways of viral infection accompanied by reduction in expression of genes underpinning the host response to viruses (Figure 2A; Table XXII in the online-only Data Supplement). Compared with men from other MSY lineages, carriers of haplogroup I1 showed alterations in gene expression in pathways responsible for fundamental defence mechanisms against pathogens including phagocytosis and lysosomal processing. Haplogroup I1 individuals also showed gene expression changes in pathways underpinning oxidative phosphorylation, mitochondrial respiration, and extracellular matrix remodeling (Figure 2A; Table XXII in the online-only Data Supplement).

In summary, this suggests that men with haplogroup I1 exhibit alterations in different molecular cascades known for their role in both early and late stages of atherosclerosis and that these gene expression changes are apparent in several types of cells and tissues of relevance to CAD.

### Haplogroup I1 Is Associated With Changes in Numerous Pathways of Relevance to Atherosclerosis in Men With CAD

We then examined the effect of haplogroup I1 on gene expression programmes in 4 relevant tissues from Swedish men affected by CAD in STAGE.^25^ We assigned all men with informative MSY phylogeny into either haplogroup I1 or other lineages and conducted a comparative transcriptome-wide analysis between these groups (Table XV in the online-only Data Supplement). There were no statistically significant differences in 3 genetic principal components between the groups (Table XXIII in the online-only Data Supplement). Our GSEA-based analysis revealed 279 pathways showing differential expression between men with haplogroup I1 and those from other lineages in at least one of 4 examined tissues (Table XXIV in the online-only Data Supplement; Figure V in the online-only Data Supplement). The haplogroup I1–associated pathways mapped onto 13 overarching biological themes, 10 of which overlapped with the themes identified in the GTEx analysis. A total of 36 pathways showed directionally consistent associations with haplogroup I1 between STAGE and GTEx analysis (Table XXV in the online-only Data Supplement). Many of these replicated I1-associated pathways (including those involved in energy production and metabolism, extracellular matrix remodeling, immunity, and response to infections) have strong relevance to atherosclerosis (Table XXV in the online-only Data Supplement). A number of additional pathways associated with haplogroup I1 in STAGE, while different in name to the ones identified in GTEx, overlapped biologically with themes and subthemes of haplogroup I1 signature identified in GTEx (Figure 2B).

We have further identified 22 pathways, exclusive to our STAGE analysis with a clear prior connection to atherosclerosis. Those mapped onto 3 overarching themes, including immune system, lipids, and coagulation and hemostasis (Figure 2B). The gene expression patterns within some of those pathways suggested upregulation of proatherogenic lipoprotein transport metabolism within the arterial wall, increased transendothelial migration of blood leukocytes, activated blood platelets, and prothrombotic phenotype within the arterial wall of men with haplogroup I1 when compared with other lineages (Table XXIV in the online-only Data Supplement; Figure 2B).

The results of secondary sensitivity analyses (conducted in STAGE to account for additional clinical cofounders) were largely consistent with the data from the primary analysis (Table XVI in the online-only Data Supplement).

In summary, we have replicated several associations between haplogroup I1 and changes in gene expression pathways underpinning fundamental mechanisms of atherosclerosis including defence against infections, innate and adaptive immunity, mitochondrial respiration, and processes of extracellular matrix remodeling. In addition, we revealed that in the presence of CAD, men with haplogroup I1 show upregulation in expression of genes from lipid, coagulation, and additional immune pathways, consistent with their increased susceptibility to atherosclerosis when compared with carriers of other Y chromosome lineages.

### Reduced Expression of Macrophage *UTY* Leads to Gene Expression Changes Partly Consistent With Those Associated With Haplogroup I1

**Figure 3.**
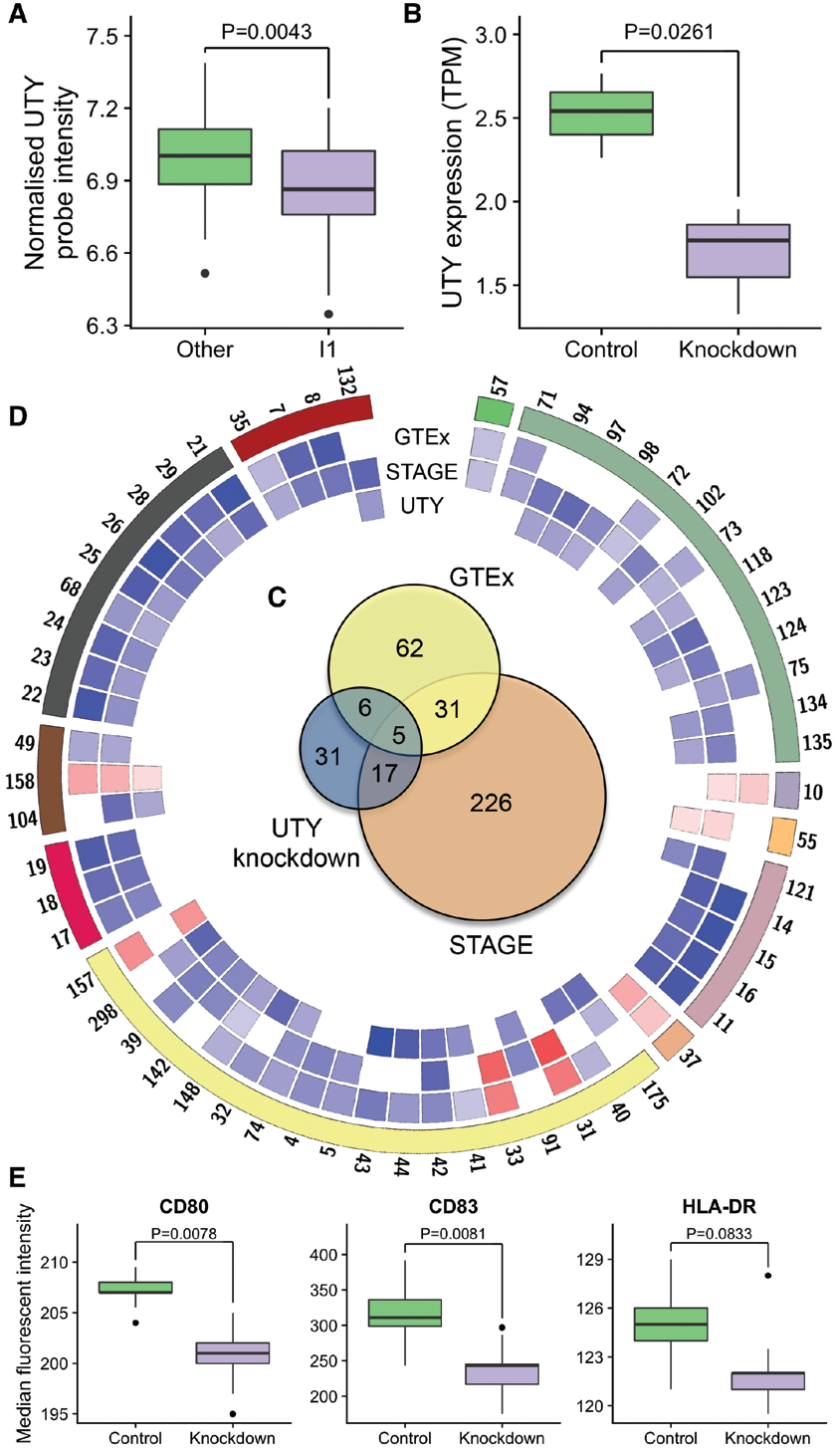
***UTY*, haplogroup I1, gene expression and immunity.** **A**, Difference in blood *UTY* expression between haplogroup I1 and other Y chromosome lineages in STAGE (Stockholm Atherosclerosis Gene Expression); *P*, level of statistical significance. **B**, Difference in *UTY* gene expression quantified by RNA sequencing between human macrophages incubated with an antisense RNA specific to *UTY* (knockdown) and macrophages incubated with a scrambled antisense RNA (control); *P*, level of statistical significance. **C**, Venn diagram showing numbers of replicating pathway results between each of the 3 analyses. **D**, Circular representation of all significant pathways overlapping between the analyses (Genotype-Tissue Expression [GTEx] project, STAGE, or *UTY* knockdown). Proceeding from the periphery inwards, numbers are unique identifiers linked to each pathway (Table XXX in the online-only Data Supplement). Colored regions represent distinct pathway themes, proceeding from vertical in a clockwise direction, these are cell contact (in green), cell cycle, cellular transport, coagulation and hemostasis, energy production and metabolism, extracellular matrix, immune system, other, transcription, translation, and viral infection (dark red). Colored tiles represent each pathway in one of 3 analyses; each analysis is labeled. The color of each tile denotes the direction of association between the expression of a pathway and haplogroup I1/*UTY* knockdown (blue, negative; red, positive). The color intensity of a tile denotes the significance of the association. **E**, Median fluorescent intensity for CD80, CD83, and HLA-DR in lipopolysaccharide-stimulated THP-1 cells after treatment with *UTY*-specific knockdown antisense RNA GapmeRs and scrambled control; *P*, level of statistical significance.

Given the key role of macrophages in atherosclerosis and the previously demonstrated effect of haplogroup I on the transcriptome of blood-derived macrophages,^5,30^ we chose this cell type as a model for in vitro experiments. We selected *UTY* as the MSY target for these studies given that it was the only one of 11 ubiquitously expressed MSY protein-coding genes associated with haplogroup I1 in blood from the STAGE study (Figure 3A; Figure VI in the online-only Data Supplement).

We then used synthetically stabilized complementary RNAs to target and experimentally reduce *UTY* mRNA expression in macrophages. We confirmed the efficacy of the experiment by both RNA sequencing and quantitative polymerase chain reaction; they showed 1.44- and 1.6-fold downregulation of *UTY* mRNA expression, respectively (Figure 3B). This drop in macrophage expression is similar to the effect of haplogroup I1 on blood *UTY* in STAGE (Figure 3A) and that of haplogroup I on macrophage *UTY* expression in our previous gene expression analysis.^30^ We also confirmed that the expression of the X-chromosome paralog (*UTX*) did not change in this experiment (*P*=0.5634; Figure VII in the online-only Data Supplement).

The experimental reduction of *UTY* expression resulted in changes of macrophage expression of 59 pathways (Table XXVI in the online-only Data Supplement); 6, 17, and 5 of the identified pathways overlapped directly with those associated with haplogroup I1 in tissues from GTEx, STAGE, or both, respectively (Figure 3C; Tables XXVII through XXX in the online-only Data Supplement). With 13 overlapping pathways mapping onto immunity (primarily: inhibition of MHC class I and II–restricted antigen presentation, as well as antigen processing), immune system was the most prominent theme intersecting the effects of haplogroup I1 and *UTY* knockdown on the transcriptome (Figure 3D; Tables XXVII through XXIX in the online-only Data Supplement). Among overlapping gene expression signals, we also observed pathways of response to viral infections and upregulation in genes within the coagulation and hemostasis pathway known for its role in atherosclerosis (Figure 3D; Tables XXVII through XXIX in the online-only Data Supplement).

To further examine the effect of *UTY* on adaptive immune responses in vitro, we experimentally reduced *UTY* expression in phorbol 12-myristate 13-acetate–treated and lipopolysaccharide-stimulated THP-1 cells and measured the expression of key molecules involved in antigen presentation and costimulation (MHC class II cell surface receptor—HLA-DR, CD80, and CD83) in these cells. We found that experimental reduction of *UTY* leads to a decrease in median fluorescent intensity for CD80 and CD83, as well as HLA-DR in these cells (Figure 3E).

Collectively, these data demonstrate a significant overlap in biological processes underpinning the gene expression profile between the experimental reduction of *UTY* in macrophages and haplogroup I1 association in tissues of relevance to atherosclerosis. This suggests that *UTY* can at least partly mediate the effect of haplogroup I1 on gene expression programmes and that some of them may be related to suppression of immunity and increased susceptibility to atherosclerosis.

## Discussion

Our study provides several novel insights into the relationship between the Y chromosome and predisposition to atherosclerosis in men. First, we identified that haplogroup I1 is the only common UK lineage of the Y chromosome associated with CAD in white British men. Second, we showed that numerous sequence variants inherited from father to son exclusively with this Y chromosome lineage are enriched in active chromatin regions within cells/tissues of relevance to atherosclerosis and as such may mediate the effect of haplogroup I1 on CAD. Third, our transcriptome-wide analyses in several human tissues revealed that the genetic effects of haplogroup I1 translate into modified gene expression programmes of direct relevance not only to atherosclerosis and the cardiovascular system but more generally to vital molecular and physiological processes. Among these, the suppression of immunity and defence against infections, changes in mitochondrial respiration, proatherogenic and prothrombotic phenotype, and alterations in extracellular matrix remodeling were the most consistent signatures of haplogroup I1 on the transcriptome. Finally, we identified one MSY gene (*UTY*) as a likely mediator of at least some of the haplogroup I1–associated effects on the human transcriptome.

The origins of haplogroup I date back to the Palaeolithic period, earlier than 20 000 years ago^12^; however, its 2 sublineages subsequently followed different histories. While haplogroup I2 (most common in south-eastern Europe) is diverse and ancient with a Palaeolithic age of ≈18 000 years, the CAD-associated haplogroup I1 is one of a number of European expansion lineages^31^ that have spread relatively recently. Its most recent common ancestor lived in the Bronze Age, only about 4000 years ago.^11^ Haplogroup I1 has a Northern European distribution,^32^ with the highest frequency in Scandinavia (≤37% in Sweden), and a declining frequency gradient southward. Popular interpretations suggest that the spread of this lineage was by migrating Vikings (https://www.eupedia.com/europe/Haplogroup_I1_Y-DNA.shtml), although no evidence has been presented for this in the scientific literature to date. Interestingly, this geographic gradient in haplogroup I1 distribution in Europe correlates with a well-known north-south difference in prevalence of CAD.^33^

Since its divergence from a common ancestor with haplogroup I2, haplogroup I1 has accumulated a large number of single genetic variants before its recent expansion. We provide comprehensive functional annotations of these variants in cells/tissues of relevance to atherosclerosis and demonstrate the enrichment of haplogroup I1–exclusive noncoding variants for transcription start sites and enhancers. Genetic variants having such characteristics are also overrepresented among autosomal signals from genome-wide association studies.^34,35^ This suggests that regulatory variants tracking with haplogroup I1 may be important for male health and disease. We did not examine whether other haplogroups of the Y chromosome individually show enrichment for active chromatin states in cells and tissues of relevance to atherosclerosis given that none of them showed individual associations with CAD. While all I1-specific variants are inherited together from father to son within a single block of DNA, their individual effects on specific cells and tissues differ; for example, some of them show operational activity only in selected tissues, whereas others appear to act in a more ubiquitous manner. We anticipate that the cumulative regulatory effect of variants in active chromatin regions is most likely to mediate the ultimate effect of haplogroup I1 on the male transcriptome, which shapes phenotypic differences between men. Because of the lack of crossing-over within the MSY, fine mapping cannot be used to dissect their individual contributions, and experimental strategies such as *CRISPR/Cas9* gene editing will instead be necessary to define their individual effects.

Our transcriptomic analyses provided compelling evidence for the global effects of haplogroup I1 on basic molecular/cellular pathways including those involved in transcription, translation, cell cycle, and metabolism. This is consistent with the increasingly recognized role of ubiquitously expressed MSY genes on RNA and protein metabolism in human tissues.^3^ However, the most apparent themes of haplogroup I1–associated pathways were those of direct relevance to atherosclerosis, in particular, the modified expression of genes in pathways responsible for antigen presentation and processing, as well as response to infections. Associations between Y chromosome and influenza A and coxsackievirus B3 infections were noted before in rodent models.^36,37^ Chronic infections including those by HIV-1 and influenza A are also increasingly recognized as a nontraditional risk factor of atherosclerosis through deregulation of immune and inflammatory response.^38,39^ The observed suppression of gene expression programmes within pathways underpinning the host response to viral infections in men with haplogroup I1 may potentially translate (through impaired defence mechanisms) into increased susceptibility, faster progression, and worse health outcomes in carriers of this haplogroup when compared with the other MSY chromosome lineages. Indeed, men with haplogroup I (of which haplogroup I1 forms a part) showed faster progression of HIV-1 infection to AIDS, higher rates of AIDS-related mortality, and worse response to retroviral therapy than men with other MSY lineages.^40^ Whether the paternally inherited suppressed response to viral infections associated with haplogroup I1 extends to other infections (which are not represented in our pathway repositories) remains unclear.

We also observed changes in gene expression within other pathways underlying almost all stages of CAD including the initiation, growth, and rupture of atherosclerotic plaque (ie, leukocyte transendothelial migration, genes involved in lipoprotein metabolism, genes encoding enzymes, and their regulators involved in the remodeling of the extracellular matrix).^41,42^ Indeed, a key leading edge gene in the majority of lipid pathways upregulated in arteries from carriers of haplogroup I1 was the apolipoprotein B gene—a marker of cholesterol-rich lipoproteins—known as the critical driver of atherosclerosis.^43^ In addition, CAD patients with haplogroup I1 showed upregulation of pathways involved in platelet aggregation and arterial thrombus formation (genes involved in ADP signaling through P2Y purinoceptor 12, genes involved in formation of fibrin clot [clotting cascade])^44^—precipitants of myocardial infarction.^45^ The gene products in these pathways are well-established targets in pharmacological management of atherosclerosis.^46^ The assessment of the extent to which the uncovered associations between haplogroup I1 and gene expression pathways are specific to one type of cell/tissue requires further studies. However, it is becoming increasingly clear that many of the disease-relevant genetic variants do not necessarily act in a tissue-specific manner, that is, in our recent analysis of variants associated with chronic kidney disease, <20% show truly kidney-specific effects on gene expression.^47^

Our data suggest that *UTY* may mediate at least some of the effects of the Y chromosome on the human transcriptome. As a minor histocompatibility antigen, *UTY* is a well-known contributor to the regulation of immune response.^48^ It also emerges that *UTY* may be involved in host response to viral infections through contributions to antigen processing and presentation.^49^ To this end, the dominance of immunity in the overlap between haplogroup I1–associated and *UTY*-related pathways is perhaps not surprising. To what extent the role of *UTY* in human immunity is driven by its function as a histone H3K27me3 demethylase remains to be established.^50^ Abundant in immune cells, H3K27me3 is considered as repressive to gene expression, and H3K27 demethylases promote transcription by removing the repressive histone mark.^51^ *UTY* shows somewhat reduced demethylase activity compared with its X-chromosome gametolog^50^ (*UTX*), but it also exhibits additional demethylase-independent chromatin remodeling activities.^52,53^ These data suggest that *UTY* can influence gene expression programmes and immunity-related processes, and its downregulation within cells of relevance to atherosclerosis may contribute to the development of at least some of the observed haplogroup I1–related molecular and clinical manifestations.

In summary, in the largest genetic association analysis to date, of 129 133 Y chromosomes, we show that a common Northern European lineage (haplogroup I1) of the MSY is enriched for multiple transcriptionally active variants and increases cardiovascular risk through proatherosclerotic reprogramming of the transcriptome in several tissues of key relevance to CAD. Our data also suggest that at least some of these changes may be mediated by downregulation of *UTY*. These data illuminate the powerful pleiotropism of the MSY on men’s cardiovascular health and disease.

## Acknowledgments

This research has been conducted using the UK Biobank Resource under application No. 15915. Access to Genotype-Tissue Expression (GTEx) project data was granted under dbGAP project 13040. Our research was supported by the high-performance computing facilities at the University of Leicester (ALICE) and Manchester (DPSF and CSF).

## Sources of Funding

The work described herein was supported by British Heart Foundation grants PG/16/49/32176 (to M. Tomaszewski), PG/12/9/29376 (to M. Tomaszewski), and RE/13/5/30177 (to T.J. Guzik and P. Maffia); National Institutes of Health R01 grant HL125863 (to J.L.M. Bjorkegren); Estonian Research Council grant PUT1036 (to P. Hallast); and European Research Council grant 726318 (to T.J. Guzik). C. Batini, P. Hallast, D. Zadik, and M.A. Jobling were supported by a Wellcome Trust Senior Fellowship grant to M.A. Jobling (087576). F.J. Charchar is supported by a National Health and Medical Research Council of Australia grant (APP 1123472).

## Disclosures

None.

## Supplementary Material
