# Supplementary material for: Human Y Chromosome Exerts Pleiotropic Effects on Susceptibility to Atherosclerosis
Source: Arterioscler Thromb Vasc Biol. 2019 Sep 5;39(11):2386–401. doi: 10.1161/ATVBAHA.119.312405 (PMC6818981; doi:10.1161/ATVBAHA.119.312405)
Supplement: Supplementary file 1 [file atv-39-2386-s001.pdf]

SUPPLEMENTAL MATERIALS

The human Y chromosome exerts pleiotropic effects on susceptibility to atherosclerosis

James M Eales, PhD[1], Akhlaq A Maan, MRes[1], Xiaoguang Xu, PhD[1],
Tom Michoel, PhD[2,3], Pille Hallast, PhD[4], Chiara Batini, PhD[5], Daniel Zadik, PhD[6], Priscilla Prestes, MSc[7], Elsa Molina, PhD[7], Matthew Denniff, MSc[8],
Juliane Schroeder, PhD[9,10], Johan LM Bjorkegren, PhD[11], John Thompson, PhD[5],
Pasquale Maffia, PhD[9,10,12], Tomasz J Guzik, MD[10,13], Bernard Keavney, MD[1,14],
Mark A Jobling, PhD[6], Nilesh J Samani, MD[8,15], Fadi J Charchar, PhD[7,8,16],
Maciej Tomaszewski, MD[1,14]*

1. Division of Cardiovascular Sciences, Faculty of Biology, Medicine and Health, University of Manchester, Manchester, United Kingdom
2. The Roslin Institute, The University of Edinburgh, Edinburgh, United Kingdom
3. Computational Biology Unit and Department of Informatics, University of Bergen, Norway
4. Institute of Biomedicine and Translational Medicine, University of Tartu, Tartu, Estonia
5. Department of Health Sciences, University of Leicester, Leicester, United Kingdom
6. Department of Genetics and Genome Biology, University of Leicester, Leicester, United Kingdom
7. School of Health and Life Sciences, Federation University Australia, Ballarat, Victoria, Australia
8. Department of Cardiovascular Sciences, University of Leicester, Leicester, United Kingdom
9. Centre for Immunobiology, Institute of Infection, Immunity and Inflammation, College of Medical, Veterinary and Life Sciences, University of Glasgow, Glasgow, UK
10. Institute of Cardiovascular and Medical Sciences, College of Medical, Veterinary and Life Sciences, University of Glasgow, Glasgow, UK
11. Department of Genetics and Genomic Sciences, Icahn Institute for Genomics and Multiscale Biology, Icahn School of Medicine at Mount Sinai, New York, United States of America
12. Department of Pharmacy, University of Naples Federico II, Naples, Italy
13. Jagiellonian University College of Medicine, Kraków, Poland
14. Division of Medicine, Manchester University NHS Foundation Trust, Manchester Academic Health Science Centre, Manchester, UK
15. NIHR Leicester Biomedical Research Centre, Leicester, UK
16. Department of Physiology, University of Melbourne, Parkville, VIC, Australia

* corresponding author
Tel: +44 161 275 0232
Fax: +44 161 275 1183
Email: maciej.tomaszewski@manchester.ac.uk
Division of Cardiovascular Sciences
The University of Manchester
Room 5.014, 5th floor
AV Hill Building
Dover Street
Manchester
M13 9PT, UK

Supplemental Figures and Figure Legends

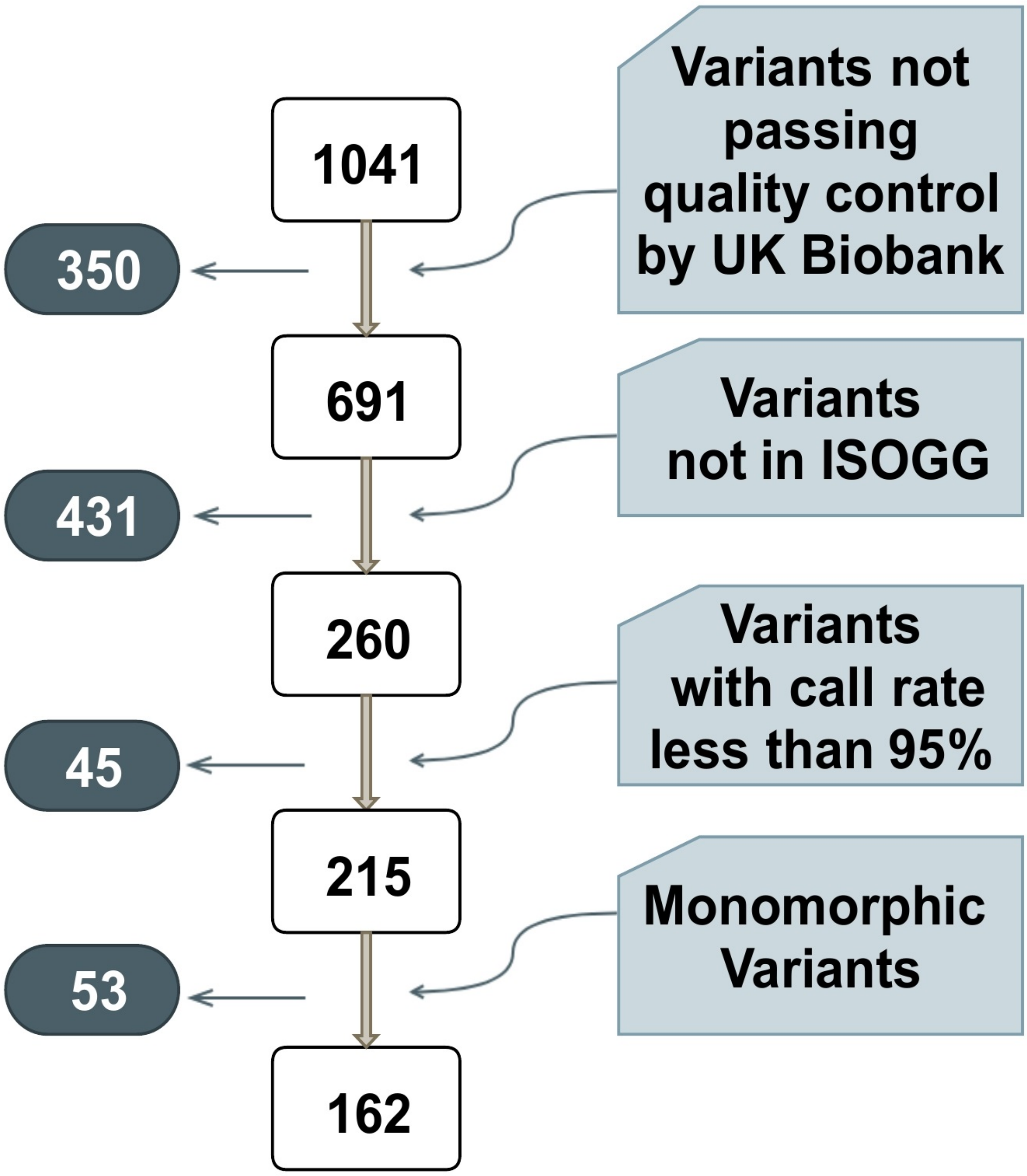

Figure I. Y chromosome genotype quality control flowchart for UK Biobank data. White boxes show number of Y chromosome single nucleotide variants at each stage of the quality control process. Light grey boxes show a brief description of each quality control step. The dark grey boxes show the number of variants removed as a result of the quality control criteria, ISOGG – International Society of Genetic Genealogy.

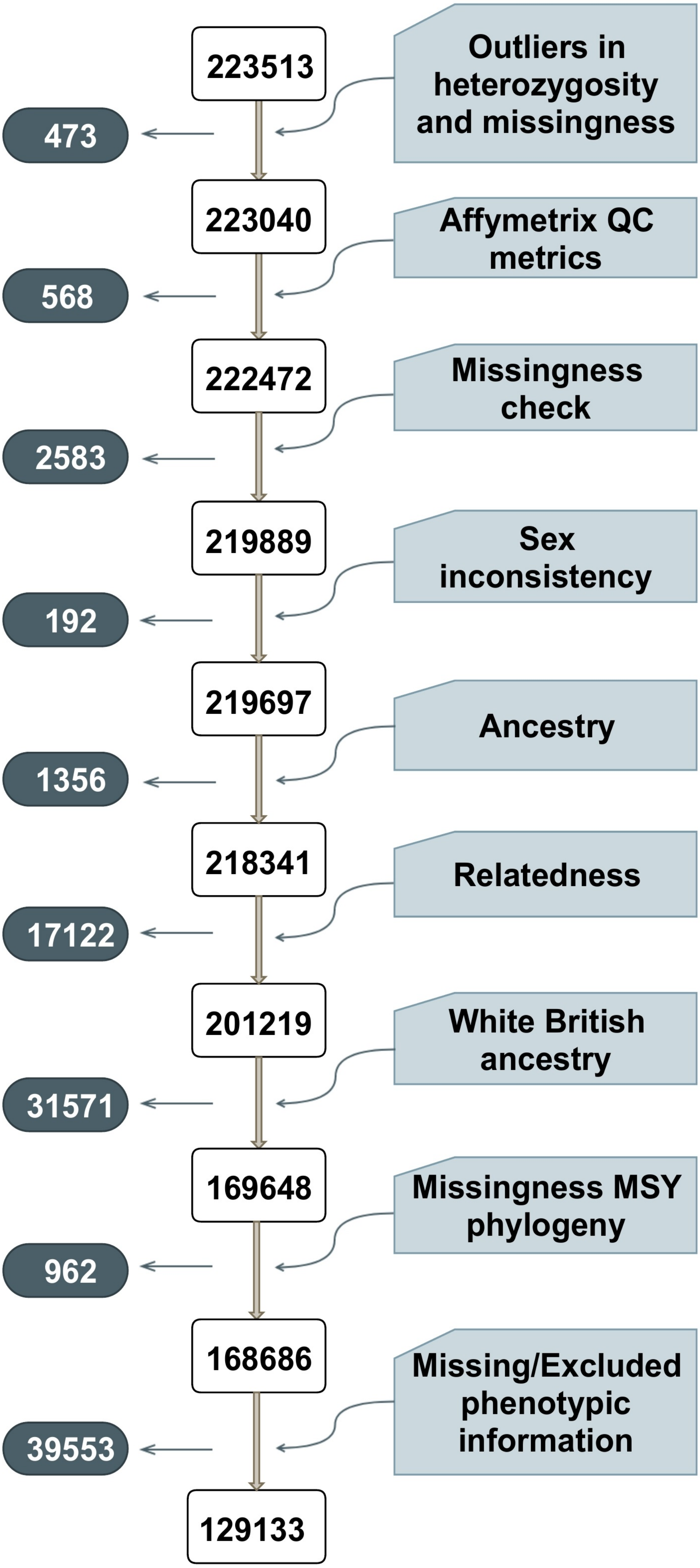
223513
Outliers in heterozygosity and missingness
473
223040
Affymetrix QC metrics
568
222472
Missingness check
2583
219889
Sex inconsistency
192
219697
Ancestry
1356
218341
Relatedness
17122
201219
White British ancestry
31571
169648
Missingness MSY phylogeny
962
168686
Missing/Excluded phenotypic information
39553
129133

Figure II. Sample quality control flowchart for UK Biobank data. The white boxes represent the number of individuals surviving each stage of the quality control process. The light grey boxes show a brief description of each step of quality control (QC) process. The dark grey boxes show the number of individuals removed as a result of each quality control step. Outliers in heterozygosity and missingness – samples identified as outliers in heterozygosity and genotype missing rates. Affymetrix QC metrics – Cluster.CR which corresponds to sample-specific call rate for each individual, computed using probesets internal to Affymetrix. Samples with Cluster.CR<97 were removed; dQC which measures the resolution of the distributions of intensity 'contrast' values, based on intensities of probe sequences for non-polymorphic genome locations. Samples with dQC<0.82 were removed. Missingness check – samples with genotype missing rate>2% were removed. Sex inconsistency – samples identified as putatively carrying sex chromosome configurations that are not either XX or XY were removed. Ancestry – ancestry outliers were removed using principal components (outliers identified if >10sd from the mean). Relatedness – for samples related to each other, only those not related (to the third degree) were selected. White British ancestry – samples were selected based on those who self-reported 'white British' and have consistent genetic ancestry based on a principal components analysis of the genotypes. Missingness MSY phylogeny – samples with missingness of Y chromosome genetic variants >2% were removed. Missing/excluded phenotypic information – men with missing information required to define coronary artery disease status (as per Figure S3) or men with outlying values required to specify the covariates for the genetic association model (as per Table I).

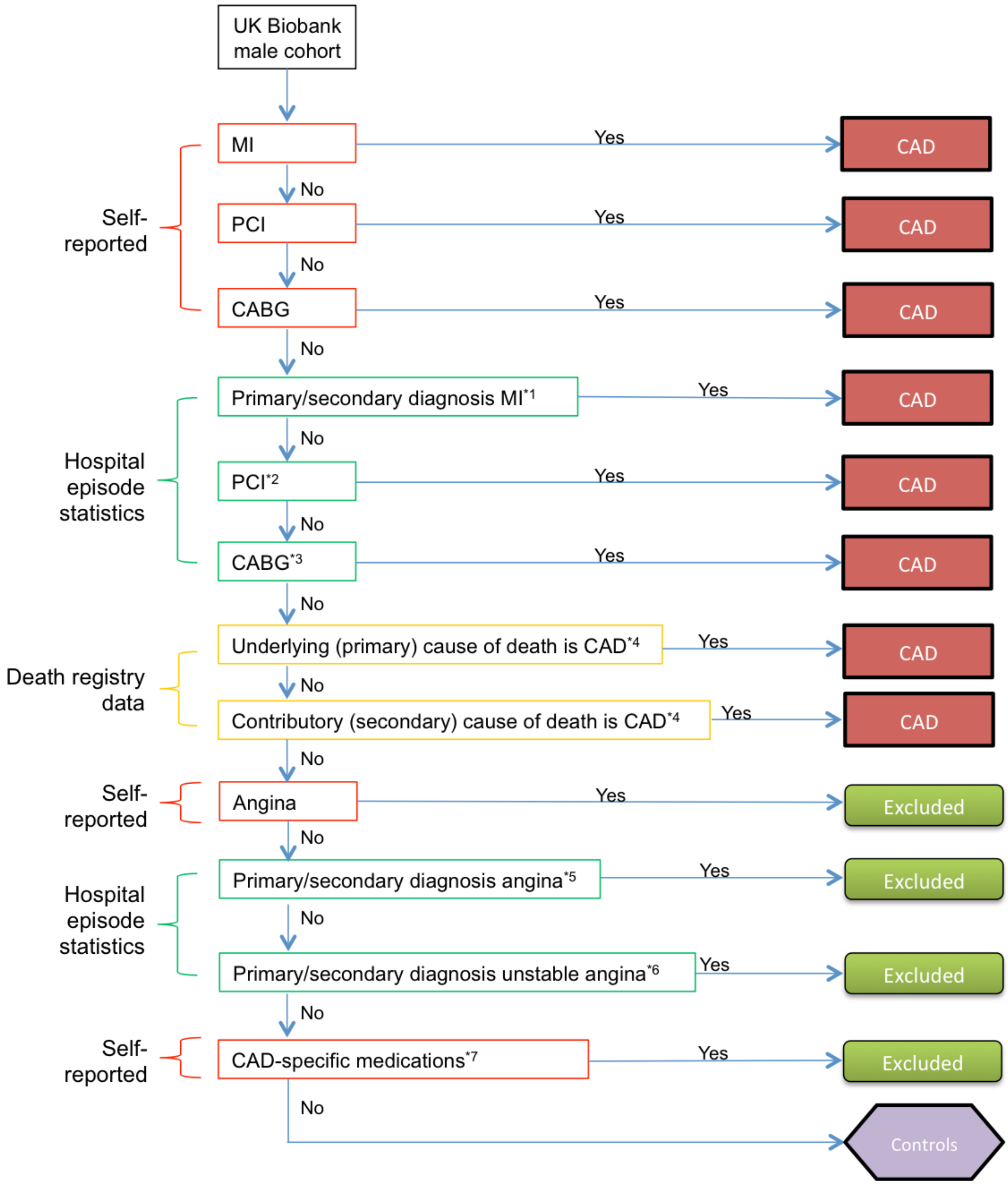

Figure III. Diagnostic decision tree for classification of UK Biobank men into patients with coronary artery disease (cases) and CAD-free controls (controls). MI – myocardial infarction, PCI – percutaneous coronary intervention, CABG – coronary artery bypass graft, ICD – International Classification of Diseases, OPCS – Office of Population Censuses and Surveys. *[1] – individuals with a history of MI were identified using ICD10 codes: I21-I23, I25.2 and ICD9 codes: 4109, 4129. *[2] – individuals with a history of PCI were identified from OPCS codes: K49, 50, 75. *[3] – individuals with a history of CABG were identified from OPCS codes K40-46. *[4] – individuals whose death was attributed to CAD were identified from ICD10 codes: I20, I21, I24, I25.1, I25.2, I25.5, I25.8, I25.9. *[5] – individuals with a diagnosis of angina were identified using ICD10 code I20.9 and ICD9 code 4139. *[6] – individuals with a diagnosis of unstable angina were identified using ICD10 codes: I20.0, I20.1, I20.8. *[7] – commonly used medications for CAD included aspirin, glyceryl trinitrate, isosorbide mononitrate/dinitrate, nicorandil.

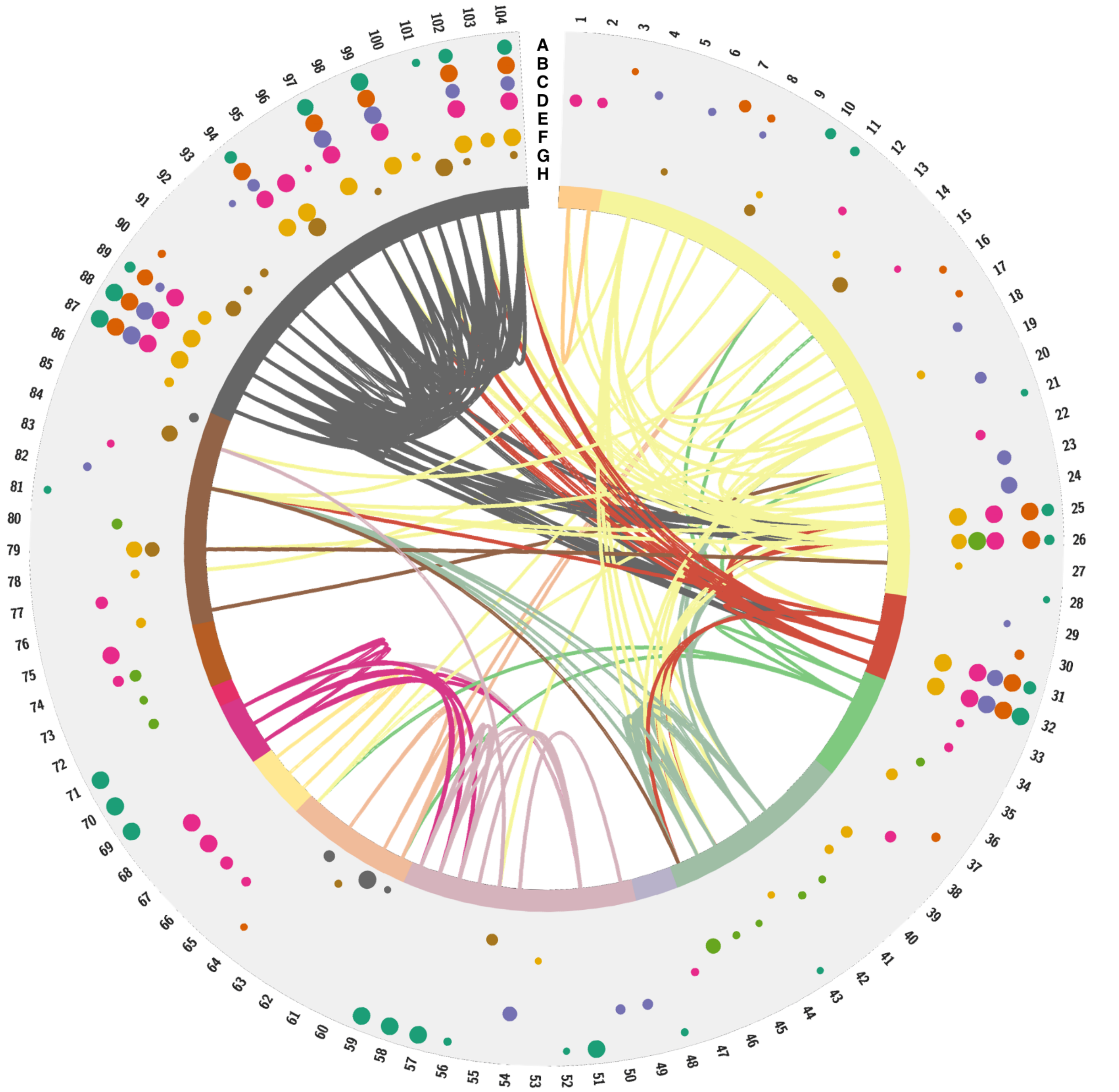

Figure IV. Overview of 104 gene expression pathways showing association with haplogroup I1 across eight GTEx tissues of relevance to atherosclerosis/CAD. The outermost circle – numerical label assigned to each pathway (full names are further provided in Table XI). The middle grey circle that contains eight tracks labelled as A-H represents -$_{\log10}$ P-values for the association of each pathway with haplogroup I1 across eight atherosclerosis and CAD-relevant tissues, including subcutaneous adipose tissue (A), visceral adipose tissue (B), aorta (C), coronary artery (D), tibial artery (E), atrial appendage tissue (F), left ventricle tissue (G), whole blood (H). The size of each dot is proportional to the magnitude of the significance for association with haplogroup I1, from the least significant (the smallest dots) to the most significant (largest dots). Functional categories of pathways are represented as coloured bars, starting from the top and proceeding clockwise they are labelled as cell contact, cell cycle, cellular transport, energy production and metabolism, extracellular matrix, haemostasis, heart disease, immune system, neurodegenerative disease, other, smooth muscle contraction, transcription and translation. The connecting lines inside the plot represent links between pathways based on a gene overlap coefficient of >0.5 and are coloured by the functional classification of the pathway.

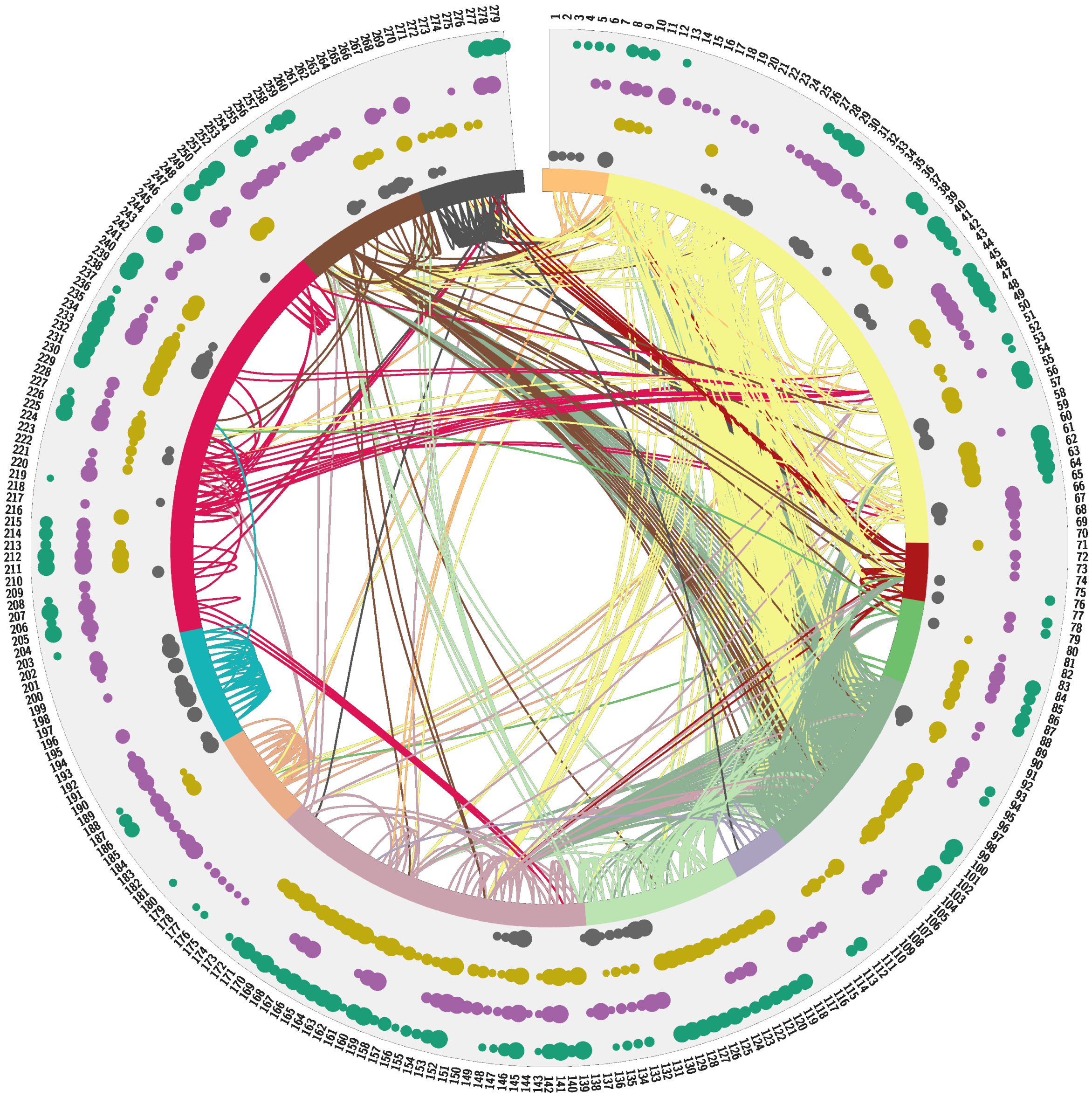

Figure V. Overview of 279 gene expression pathways showing association with haplogroup I1 across four STAGE tissues of relevance to atherosclerosis and CAD. The outermost circle – numerical label assigned to each pathway (full names are further provided in Table XX). The middle grey circle that contains four tracks labelled as A-D represents -$_{\log10}$ P-values for the association of each pathway with haplogroup I1 across four atherosclerosis and CAD-relevant tissues, including atherosclerotic artery wall (A), subcutaneous fat (B), visceral fat (C) and whole blood (D). The size of each dot is proportional to the magnitude of the significance for association with haplogroup I1, from the least significant (the smallest dots) to the most significant (largest dots). Functional categories of pathways are represented as coloured bars, starting from the top and proceeding clockwise they are labelled as coagulation and haemostasis, immune system, viral infection, cell contact, cell cycle, cellular transport, DNA, energy production and metabolism, extracellular matrix, lipids, other, transcription and translation. The connecting lines inside the plot represent links between pathways based on a gene overlap coefficient of >0.75 and are coloured by the functional classification of the pathway.

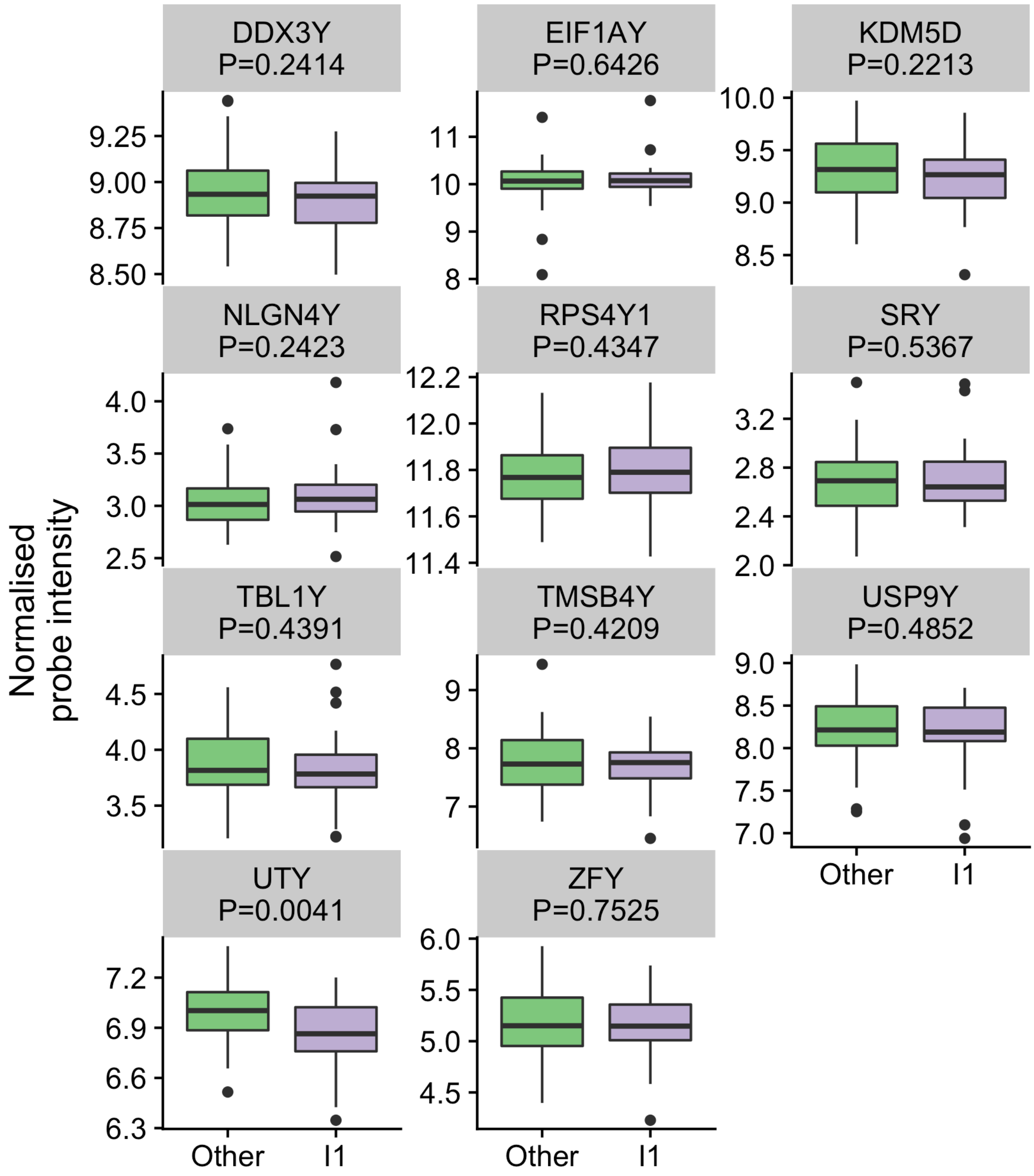

Figure VI. Boxplot showing differences in normalised probe intensity values from the genotyping array for 11 X-degenerate ubiquitously expressed MSY genes between haplogroup I1 and other Y chromosome lineages in blood from STAGE. P-value – level of statistical significance.

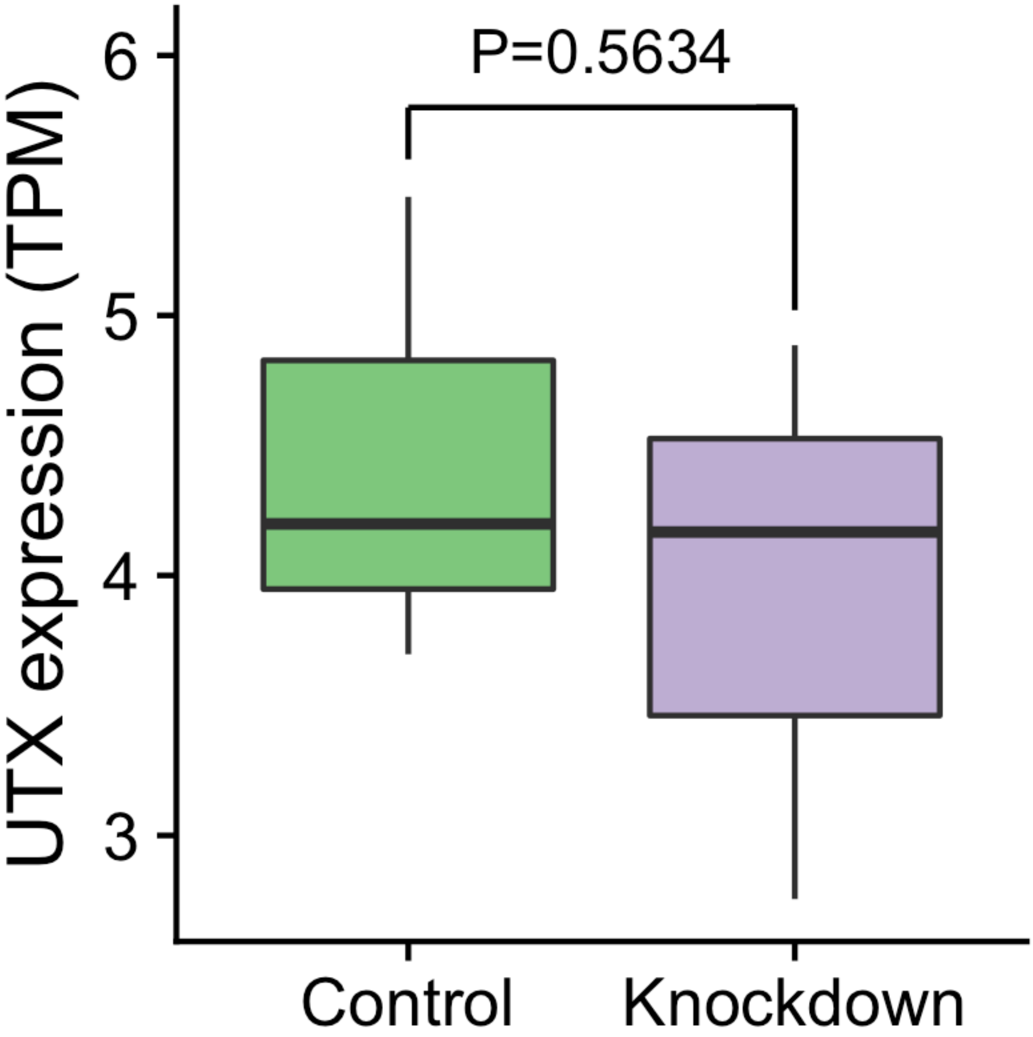

Figure VII. Analysis of difference in blood macrophage UTX expression (quantified by RNA-sequencing) between cells incubated with an antisense RNA specific to UTY ("knockdown") and cells incubated with a scrambled antisense RNA ("control"). P-value – level of statistical significance.

Major Resources Tables

Animals (in vivo studies)

Species	Vendor or Source	Background Strain	Sex

Animal breeding

	Species	Vendor or Source	Background Strain	Other Information
**Parent - Male**				
**Parent - Female**				

Antibodies

Target antigen	Vendor or Source	Catalog #	Working concentration	Lot # (preferred but not required)
CD14 (Pe-Cy7)	Thermofisher		Diluted from stock 1 : 20	
CD80 (FITC)	Thermofisher		Diluted from stock 1 : 20	
CD83 (APC)	Thermofisher		Diluted from stock 1 : 20	
HLA-DR (e450)	Thermofisher		Diluted from stock 1 : 20	

Cultured Cells

Name	Vendor or Source	Sex (F, M, or unknown)
THP-1	Lab-stock	Male
